# Human Milk Composition and Nutritional Status of Omnivore Human Milk Donors Compared with Vegetarian/Vegan Lactating Mothers

**DOI:** 10.3390/nu15081855

**Published:** 2023-04-12

**Authors:** Noelia Ureta-Velasco, Kristin Keller, Diana Escuder-Vieco, Javier Fontecha, María V. Calvo, Javier Megino-Tello, José C. E. Serrano, Carmen Romero Ferreiro, Nadia Raquel García-Lara, Carmen R. Pallás-Alonso

**Affiliations:** 1 Department of Neonatology, Research Institute i+12, 12 de Octubre University Hospital, Complutense University of Madrid, 28041 Madrid, Spain; 2 “Aladina-MGU”—Regional Human Milk Bank, Research Institute i+12, 12 de Octubre University Hospital, 28041 Madrid, Spain; 3 Food Lipid Biomarkers and Health Group, Institute of Food Science Research (CIAL), CSIC-UAM, 28049 Madrid, Spain; 4 Department of Experimental Medicine, Faculty of Medicine, University of Lleida, 25008 Lleida, Spain; 5 Scientific Support Unit, Research Institute i+12, 12 de Octubre University Hospital, 28041 Madrid, Spain; 6 Faculty of Health Sciences, Universidad Francisco de Vitoria, Pozuelo de Alarcón, 28223 Madrid, Spain

**Keywords:** breast milk, human milk bank, vegetarian, diet, nutritional status, lipid profile, vitamins, minerals, vitamin B12, docosahexaenoic acid

## Abstract

Women of childbearing age in Western societies are increasingly adopting vegetarian diets. These women are sometimes rejected as milk donors, but little about the composition of their milk is known. The present study aimed to compare the intake, nutritional status, and nutritional composition of human milk from omnivore human milk donors (Donors) and vegetarian/vegan lactating mothers (Veg). Milk, blood, and urine samples from 92 Donors and 20 Veg were used to determine their fatty acid profiles, as well as vitamins and minerals. In a representative sample of both groups, we also determined the lipid class profile as a distribution of neutral and polar lipids, the molecular species of triacylglycerols, and the relative composition of phospholipids in their milk. A dietary assessment was conducted with a five-day dietary record (while considering the intake of supplements). We highlight the following results, expressed as the mean (SE), for the Veg vs. Donors: (1) Their docosahexaenoic acid (DHA) intake was 0.11 (0.03) vs. 0.38 (0.03) g/day; the plasma DHA was 0.37 (0.07) vs. 0.83 (0.06)%; and the milk DHA was 0.15 (0.04) vs. 0.33 (0.02)%. (2) Their milk B12 levels were 545.69 (20.49) vs. 482.89 (4.11) pM; 85% of the Veg reported taking B12 supplements (mean dose: 312.1 mcg/day); and the Veg group showed no differences with Donors in terms of total daily intake or plasma B12. (3) Their milk phosphatidylcholine levels were 26.88 (0.67) vs. 30.55 (1.10)%. (4) Their milk iodine levels were 126.42 (13.37) vs. 159.22 (5.13) mcg/L. In conclusion, the Vegs’ milk was shown to be different from the Donors’ milk, mainly due to its low DHA content, which is concerning. However, raising awareness and ensuring proper supplementation could bridge this gap, as has already been achieved for cobalamin.

## 1. Introduction

A mother’s own milk is the gold standard for the feeding of preterm and full-term infants. The World Health Organization (WHO) recommends exclusive breastfeeding for the first 6 months of life and its continuation during the introduction of complementary foods, up to 2 years or longer [1]. For preterm infants, donor human milk (DHM) is the preferred feeding strategy when their own mother’s milk is not available [2,3,4].

Despite the importance of human milk in infant nutrition, there is a lack of robust knowledge about its nutritional composition; moreover, this is even more evident in DHM. A recent systematic review highlighted the lack of available information on the vitamin and mineral composition of DHM. Furthermore, the findings in maternal milk should not be generalized to DHM due to additional sources of variation conditioned by the processes that DHM undergoes (i.e., expression, freezing, storage, pooling, mixing, multiple container transfers, and pasteurization) [5]. Premature infants have increased nutritional needs, so studying the nutritional content of DHM and the factors that may influence it should be mandatory.

Among the many factors that influence the composition of human milk, one of the most interesting is the mother’s diet, as it is a modifiable factor that can be improved with health advice. However, the impact of the maternal diet on the nutritional profile variations in human milk between women is not yet fully understood, most likely because of the fact that there are many factors that modify the composition of the milk, not only those related to the characteristics of the breastfeeding woman, such as genetics, diet, lifestyle, etc., but also related to breastfeeding itself, such as the type and time of expression, the stage of lactation, etc. [5,6,7,8]. In addition, studies have generally focused on a few nutrients and have not assessed the full nutritional profile of milk.

In the last few years, there has been a renewed effort to better understand the relationship between the maternal diet, nutritional status, and human milk quality. This has been conducted not only in resource-poor countries, but also in resource-rich settings, since modern trends in diet and lifestyle may compromise human milk nutrient levels [9,10].

Moreover, women of childbearing age are increasingly adopting vegetarian or vegan diets in Western societies. However, despite the numerous health benefits, vegetarians without proper dietary advice are at a high risk of inadequate intake of and an inadequate status for several nutrients, mainly vitamin B12, vitamin D, iron, iodine, zinc, calcium, selenium, and docosahexaenoic acid (DHA). Additionally, the more restrictive the vegetarian diet is, the greater the risk of dietary inadequacy [11,12]. Consequently, human milk banks have been faced with the challenge of determining whether these women are suitable to be milk donors, with the aim of ensuring the safety and quality of DHM via donor selection processes [13].

Following the recommendations of the European Milk Bank Association, a vegan diet with an adequate supplementation of vitamin B12 is not an exclusion criterion for donor candidates [13]. Nevertheless, certain countries do not accept milk donations from vegans, regardless of their vitamin B12 supplementation status [12]. According to the American Dietetic Association, the breast milk of vegetarian mothers with an appropriately planned diet is similar in its composition to that of non-vegetarians, except with respect to fatty acid (FA) concentration [14].

However, only a few studies have determined the macronutrients, FAs, vitamin B12, folate, and minerals in the milk of women who follow a vegetarian/vegan diet [12,15], whereas other important nutrients have not been assessed, including fat-soluble vitamins, vitamin C, many of the B group vitamins, and other lipid compounds different from FAs.

Finally, we could not find any study focused on comparing the human milk nutritional composition of DHM with that of vegetarian women.

The objective of the present study is to compare the maternal intake, nutritional status, and nutritional composition (i.e., macronutrients, water-soluble and fat-soluble vitamins, minerals, FA profile, lipid class prolife, molecular species of triacylglycerols, and the relative composition of phospholipids) of human milk from omnivore human milk donors and vegetarian/vegan lactating mothers.

## 2. Materials and Methods

### 2.1. Study Design and Subjects

This cross-sectional, observational study was conducted at the Regional Human Milk Bank Aladina MGU (RHMB) at “12 de Octubre” University Hospital in Madrid, Spain. The subjects of study were as follows: (1) human milk donors with an omnivore diet and with full-term infants (Donors) who donated milk at least once in the last 2 months to the RHMB; and (2) healthy vegetarian/vegan lactating mothers (Veg) with a milk expression routine who were lactating 3 weeks or more postpartum. Vegetarians were defined as women who did not consume any kind of meat or fish, but did consume eggs and/or milk, including ovo-vegetarians, lacto-vegetarians, and ovo-lacto-vegetarians. Vegans were defined as those women who also excluded any animal-derived foods, such as eggs, dairy products, honey, beeswax, gelatin, and other animal-derived ingredients.

Participants were recruited between August 2017 and February 2020.

The study procedure was endorsed by the “12 de Octubre” University Hospital Clinical Research Ethics Committee (protocol code 15/269) and all participants provided written informed consent, which could be withdrawn at any time. Furthermore, the study was conducted in accordance with the Declaration of Helsinki.

#### 2.1.1. Sample Size Calculation

The present work is part of a larger study in which 3 groups of women were studied: (1) human milk donors, (2) vegetarian/vegan lactating mothers, and (3) lactating mothers of very preterm and/or very-low-birth-weight infants—i.e., ≤32 weeks gestational age and/or ≤1500 g—who were admitted to the neonatal unit of the “12 de Octubre” University Hospital at the time of the study. One of the main aims of this work was to study the correlations between the intake, nutritional status, and human milk nutritional composition of the mothers. For this purpose, the sample size was calculated to guarantee the generalizability of the results. In this sense, considering the need for at least 10 cases per predictor and an additional 5% for possible losses, a sample of 115 participants was estimated for the models of the global study, in which a total of 10 predictors of clinical interest were included. This involved the recruitment of at least 85 milk donors, 15 healthy lactating women on a vegetarian diet, and 15 mothers of preterm infants. For this study, we selected milk donors with an omnivorous diet and a full-term infant as a group which is more comparable to that of vegetarian mothers.

#### 2.1.2. Study Protocol

The study participants were contacted and provided with detailed information on the objectives of the study and the procedures to be carried out. Donors’ contact details were provided by the RHMB. Vegetarians/vegans were advised about the study by the pediatricians of the primary care centers in the “12 de Octubre” University Hospital’s health area, as well as through advertisements placed on online communities that were focused on breastfeeding or vegetarianism, on websites dedicated to vegetarians, or in vegetarian food shops.

An early morning meeting at the RHMB was arranged for each of the women who agreed to participate in the study. At this appointment (day 0), the participant’s blood and urine samples for biochemical studies, somatometric measurements, informed consent for the study, health and socio-demographic survey, and food frequency questionnaire (FFQ) were collected. The participants provided a sample of their first urine that morning. If they forgot, a sample was obtained at the RHMB while the women were still fasting. The urine was transferred to two 10 mL test tubes and frozen at −80 °C. A fasting blood sample was collected in four tubes of EDTA. The blood tubes were wrapped in foil to protect the light-sensitive nutrients and then refrigerated at −4 °C until they were processed within the following 2 h.

Women were weighed and measured, and their body mass index (BMI) was also calculated (using an electronic medical scale with BMI function: Model 799 with measuring rod Model 220, CE approved—Class III, brand Seca^®^, Hamburg, Germany).

The health and socio-demographic survey included information about mother’s age, country of birth, educational level, and work situation, as well as her details regarding food exclusion; dietary changes in the previous 2 years; consumption of iodized salt; supplementation during pregnancy and lactation; alcohol and tobacco consumption; physical activity; season during the study; diseases; medication intake; offspring; pregnancy; childbirth; lactation; milk expression; weight prior to last pregnancy; and weight gain during pregnancy. The participants also provided information regarding sex and somatometric values, as well as diseases of their breastfed infants.

The FFQ was adapted to the vegetarian diet and assessed the daily/weekly/monthly intake of the following: (1) milk; (2) other dairy products; (3) meat and meat products; (4) fish; (5) eggs; (6) fruits, e.g., fresh juices and dried fruits; (7) raw vegetables; (8) cooked vegetables; (9) legumes (including peanuts, soya beans, and derivates) and seitan; (10) bread, pasta, rice, and other cereals (including rice and oat drinks); (11) nuts and seeds; (12) sweets; and (13) fats and oils (olive, sunflower, sesame, soya, coconut, etc.). A photo atlas was used to determine the serving weight (grams) [16]. The recorded weight of food was used to calculate the daily or weekly ingested rations of each food considering the weight of each ration, as proposed by the Spanish Society of Community Nutrition [17] and adopted by the Spanish Ministry of Health [18].

At least one of the researchers reviewed all the data and assisted the participants in completing the questionnaires, provided the materials necessary for maintaining their dietary record and performing their milk collection, and explained the study procedure again. Written information as well as a telephone number was provided to the participants for those with further questions.

Within the following 15 days after their visit to the RHMB, participants were asked to choose 6 days in a row to conduct the second part of the study. During the first 5 days (days 1 to 5) they filled in a dietary record. In parallel, from days 2 to 5, they collected a milk sample of 25 mL from each expression (at least one per day) for vitamin and mineral studies. On the sixth day (day 6), they collected a complete milk sample from one of their breasts for the lipid studies. Participants were requested to bring their dietary records and milk samples to the RHMB within the following 15 days after study’s completion.

**Figure 1:**
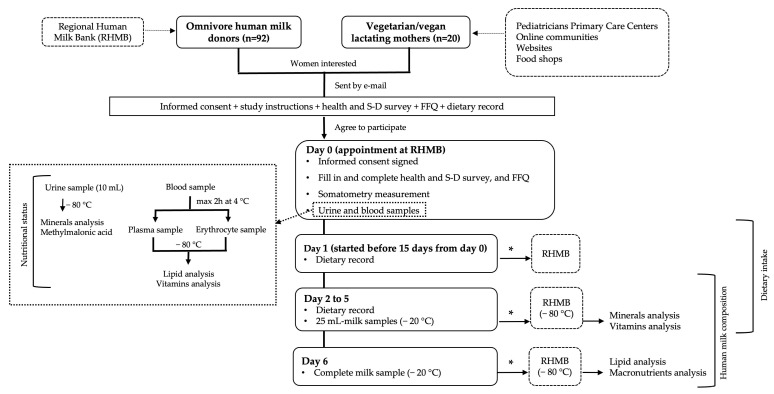
Flow chart of the study’s protocol. * Delivered to the RHMB within the following 15 days. Abbreviations: S-D, socio-demographic; FFQ, food frequency questionnaire; RHMB, Regional Human Milk Bank.

The vitamins and lipids were determined in the plasma, erythrocytes, and milk. Minerals were determined in milk and urine. Vitamins and minerals in milk were detected on each of the four consecutive days when the participants expressed milk for this purpose. Figure 1 shows the study’s protocol.

### 2.2. Blood Samples Processing

Blood samples were processed at the Mitochondrial Diseases Laboratory of the “12 de Octubre” University Hospital within two hours of blood extraction. The tubes were protected from light by being wrapped in foil throughout the process. Plasma was obtained after centrifuging blood samples in EDTA tubes at 1200× *g* for 10 min at 4 °C, which were then aliquoted into 5 opaque 1 mL Eppendorf^®^ (Hamburg, Germany) tubes for the vitamins study and 1 transparent 1 mL Eppendorf^®^ tube for the lipid study. Erythrocytes samples were obtained following the protocol for washing red blood cells described by López Martínez et al. in the Technical Document of the Spanish Society of Clinical Biochemistry and Molecular Pathology [19], similar to that described by Klem et al. [20]. Briefly, after collecting the plasma, a 0.9% saline solution was added until the tube containing the erythrocyte pellet was filled. Then, the contents were mixed by inverting the tube several times. After centrifugation for 10 min at 1200× *g*, the supernatant was removed with a Pasteur pipette. This process was repeated two more times and finally the washed erythrocytes were aliquoted into 4 opaque 1 mL Eppendorf^®^ tubes for the vitamins study and 1 transparent 1 mL Eppendorf^®^ tube for the lipid study.

The aliquots obtained were stored and frozen at −80 °C in the RHMB.

### 2.3. 5-Day Dietary Record

For five consecutive days (including at least one weekend or holiday), the participants were asked to record their diet at the time that their food and beverages were consumed, including vitamin and mineral supplements, medicines, and salt (specifying whether it was iodized). Whenever possible, the food taken was weighed; household measures, such as cups or spoons, could be used if an exact measurement of weight was not possible. The participating women were also encouraged to provide ingredients and nutritional information if they consumed processed food.

DIAL^®^ software (DIAL.EXE Version 3, February 2014, Alce Ingeniería, Madrid, Spain) was employed to calculate the energy and nutrients provided by the food [21]; this tool uses the “Food Composition Tables” published by the Department of Nutrition of the Complutense University of Madrid in 2010. The food items not included in the program were created based on the information provided by participants (i.e., food packages) or databases such as the Spanish Food Composition Database [22] or the Food Data Central of the U.S. Department of Agriculture [23]. The caloric and lipid profiles, the healthy eating index (HEI), and the daily intake values for energy and for each nutrient were obtained. The nutrients provided by the intake of vitamin or mineral supplements were taken into account in the calculation of the daily nutrient intake.

Reference values for the caloric and lipid profiles regarding the total energy of the diet were as follows: (1) 10–15% for proteins, (2) 20–35% for lipids, (3) more than 50% for carbohydrates, (4) less than 10% for saturated fatty acids (SFAs), and (5) 4–10% for polyunsaturated fatty acids (PUFAs). These values were obtained by following the nutritional objectives for the Spanish population [24]. The daily nutrient intake was compared with the dietary reference intake (DRI) of the Institute of Medicine (IOM) [25,26,27,28,29,30] and the dietary reference values of the European Food Safety Authority (EFSA) for nursing mothers [31]. The intake of PUFAs was compared with the IOM recommendations [29] and those of the Food and Agriculture Organization [32]. The diet quality evaluation of the participants was based on the HEI and was categorized as follows: (1) >80 excellent, (2) 71–80 very good, (3) 61–70 good, (4) 51–60 acceptable, and (5) 0–50 inadequate [33,34].

### 2.4. Milk Sample Collection and Processing

The human milk samples for vitamin and mineral assessment were collected at home over four consecutive days (at least once per day), starting one day after the first day of dietary recording. The purpose was to reproduce the conditions of their routine in terms of daily milk pumping. Therefore, participants were asked not to change their expression routine, no request was made for a time schedule, and the same transparent glass containers were used as for donation. Milk could be expressed from one or both breasts, the only requirement being a complete emptying of the breast. From each milk extraction, 25 mL was collected using a sterile syringe after gently shaking the container, and the milk was then placed in an additional sterile feeding bottle and frozen at −20 °C. For the lipid assessment, an entire sample on the sixth day via the complete expression of one breast was collected and frozen at −20 °C, without manipulation. Samples were labelled with a participant code, the date and time of expression, and the total volume expressed.

The storage time of the milk samples at the subjects’ homes averaged 10.5 days (minimum 1 day, maximum 45 days). The home milk-freezing time was slightly longer in the Donors group (mean 11.8 in the Donors group vs. 9.1 in the Veg group, *p* < 0.001).

Participants transported the frozen milk samples in portable coolers with cold packs and delivered them to the RHMB, where they remained frozen at −20 °C until processing. Then, every 25 mL milk bottle was thawed in a 40 °C water bath, gently shaken to homogenize the milk, and then divided into twenty 1 mL aliquots in transparent Eppendorf^®^ tubes. If a woman had expressed more than one milk sample per day, then, after thawing the milk, the different samples from the same day were mixed, shaken gently to homogenize them, and 20 1 mL aliquots were obtained from the mixture. Milk from different days was not mixed. Each aliquot was labelled with the code of the participant and the day of expression (day 2 to 5) and then frozen at −80 °C. The complete extraction on day 6 was frozen without manipulation.

### 2.5. Sample Storage and Shipment

The plasma, red blood cells, urine, and milk samples were stored frozen at −80 °C in the RHMB. Samples were sent on dry ice to their respective laboratories for analyses, where they were also stored at −80 °C.

### 2.6. Lipid Analysis

Lipid analyses were conducted by the Food Lipid Biomarkers and Health Group at the Institute of Food Science Research (CIAL), CSIC-UAM. The FA profile was studied in the erythrocytes, plasma, and milk from all the donors and the vegetarians/vegans included in the study. The lipid class profile as distribution of neutral and polar lipids, the relative composition of phospholipids (PLs), and the molecular species of triacylglycerols (TAGs) were determined in the milk samples from 18 Veg, as well as from a randomly selected subgroup of 19 Donors. 

#### 2.6.1. Fat Extraction

The fat from the human milk samples was extracted following the method described by Löfgren et al., (2012) [35], with some modifications based on the optimization of the solvent/ratio sample as described by García-Serrano et al., (2020) [36]. The lipid extracts were filtered through 0.45 μm PVDF filters, collected in amber vials, dried under a nitrogen stream, and then stored at −35 °C until further chromatographic analysis.

#### 2.6.2. Chromatographic Analyses

##### Separation and Quantification of Lipid Classes by HPLC Evaporative Light Scattering Detector (ELSD)

The separation of the lipid classes was accomplished in an HPLC system (model 1260; Agilent Technologies Inc. Palo Alto, CA, USA) coupled with an ELSD (SEDEX 85 model; Sedere SAS, Alfortville Cedex, France) while using prefiltered compressed air as the nebulizing gas at a pressure of 350 kPa at 60 °C; in addition, the gain was set at 3. Two columns in series (250 × 4.5 mm Zorbax Rx-SIL column with 5-μm particle diameter; Agilent Technologies Inc.) and a pre-column with the same packing were used. Before analysis, samples were dissolved in dichloromethane (5 mg/mL) and 50 μL was injected after column equilibration at 40 °C. The solvent gradient was conducted as detailed in Castro-Gómez et al., (2017) [37]. Both samples and standards were analyzed under the same conditions, using solvents that were freshly prepared.

#### Determination of Fatty Acid Methyl Esters (FAMEs) by GC-MS

FAMEs were directly prepared from the plasma and erythrocyte samples (200 μL), without prior lipid extraction. However, in the case of the milk samples, 10 mg of the lipid extracts previously obtained was used. Two independent derivatization processes were carried out for each sample following the direct acid–base methylation method, as described by Castro-Gomez et al., (2014) [38]. Prior to methylation, tritridecanoin (13:0 TAG, 75 μL; 1.0 mg/mL) was added to the samples as an internal standard.

FAMEs analysis was performed in an Agilent 6890 series gas chromatograph which was coupled to an Agilent 5973 series mass spectrometer (Agilent Technologies Inc., Palo Alto, CA, USA). A CP-Sil 88 fused-silica capillary column (100 m × 0.25 mm × 0.2 mm film thickness; Chrompack, Middelburg, The Netherlands) was employed for chromatographic separation as described by Calvo et al., (2020) [39]. The temperature program was as follows: 1 min at 100 °C; first ramp was 7 °C/min up to 170 °C; the temperature was held for 55 min; then it was increased at 10 °C/min up to 230 °C; and then it was held for 33 min. The total time for the chromatographic run was 105 min. Helium was used as a carrier gas with a column inlet pressure of 30 psi. The injection volume was 1 μL and the split ratio was 1:25. The MS detector conditions were as follows: transfer line temperature of 250 °C; ion source temperature of 230 °C; and quadrupole temperature of 150 °C. The MS was operated under an electron impact ionization at 70 eV. It was then used in total ion current (TIC) mode to scan the mass range from 40 to 500 m/z. Anhydrous milk fat (reference material BCR-164; Fedelco Inc., Madrid, Spain) was assayed to determine and calculate the response factor for FAMEs. FAMEs data analysis was presented as weight/weight percentages. In total, 15 FAMEs in erythrocytes, 14 in plasma, and 30 in milk were quantified, with a chain length of 6–22 carbon atoms. In general terms, the limit of quantitation was 1.8 ppm. Plasmalogens, as their dimethylacetal derivatives (DMA), were also determined during the GC/MS analysis.

#### Determination of TAG Molecular Species by GC-FID

TAG molecular species were quantified according to their number of carbon atoms (CN). Analyses were performed on a Clarus 400 GC (PerkinElmer Ltd., Beaconsfield, UK), which was equipped with an automatic split/splitless injector and a flame ionization detector. An Rtx-65TAG fused silica capillary column (30 m × 0.25 mm i.d. × 0.1-μm film thickness; Restek Corp., Bellefonte, PA, USA) was used. Experimental chromatographic conditions were the same as those published by Fontecha et al., (2006) [40]: 120 °C, held for 30 s; 10 °C/min to 220 °C and held for 30 s; and then 6 °C/min to 350 °C and held for 30 min. The injector and flame ionization detector temperatures were 355 and 370 °C, respectively. Helium was used as the carrier gas (172 kPa). Lipid extracts were dissolved in dichloromethane (20 mg/mL) and the injection volume was 0.5 μL. For the qualitative and quantitative analysis of TAGs, the response factors were calculated using an anhydrous milk fat (reference material BCR-519; EU Commission, Brussels, Belgium; purchased from Fedelco Inc., Madrid, Spain), with a known TAGs composition and glyceryl trinanoate as the internal standard (9:0 TAG, 100 μL; 1 mg/mL).

### 2.7. Vitamins and Minerals Analysis

The vitamins and minerals analyses were conducted by NUTREN-Nutrigenomics Group of the Department of Experimental Medicine of the University of Lleida, Spain.

#### 2.7.1. Minerals

Minerals in breastmilk were determined following the methodology described by Huynh et al., (2015) [41]. Briefly, 1 mL of homogenized breast milk was placed into labeled 50 mL polypropylene tubes, 5 mL 8% TMAH and 0.75 mL pure water were then added to each of the tubes using the diluter, and then the tubes were recapped. Samples were mixed by shaking/vortexing at low speed and were allowed to stand overnight in a fume hood at room temperature. On the following day, samples were mixed again by shaking/vortexing and digested at 90 °C for 1 h using the heating block system. Samples were mixed by shaking/vortexing at least twice during the incubation period to ensure complete digestion. The tubes were then removed from the heating block and cooled at room temperature. Then, 2.25 mL of pure water was added to all tubes using the diluter and the volume was increased to 40 mL by the addition of 30 mL of high-purity water. The tubes were tightly recapped and shaken/vortexed until thoroughly mixed. Then 5–10 mL of each digested solution was filtered and transferred into 1.5 mL tubes, prior to ICPMS analysis. The minerals in the urine samples were directly determined after a 20-fold dilution. Briefly, 0.25 mL of urine was added to 4.75 mL of an aqueous solution containing 2% 1-butanol, 0.05% EDTA, 0.05% Triton X-100, and 1% NH_4_OH. Determination was then carried out using an Agilent 7500ce inductively coupled plasma mass spectrometry system, consisting of an integrated sample introduction system (ISIS) unit plus a CETAC ASX-510 auto-sampler (Agilent Technologies, Mulgrave, Australia), which was equipped with a Ceramic VeeSpray nebulizer (Glass Expansion Pty. Ltd., Melbourne, Australia).

#### 2.7.2. Water-Soluble Vitamins and Vitamin-B12-Associated Biomarkers

All blood and milk handling in the laboratory was carried out in subdued light to minimize the effects of the light on the vitamin concentrations.

The amount of vitamin B12 in the milk was determined by an immunoassay determination in the deproteinized samples. Briefly, samples were centrifuged to separate and eliminate the fat. For 500 µL of breastmilk (without fat), 100 µL of sodium acetate (pH4, 1 M), 50 μL of 1% potassium cyanidin, and 2.2% of pepsin were added. The samples were incubated at 37 °C with an automated shaker (120 rpm) for protein digestion for 3 h. Further, the samples were heated at 90 °C to ensure a quantitative conversion of all forms of vitamin B12 to cyanocobalamin. After cooling, the samples were filtered and analyzed by a competitive immunoassay method (Ref. 33000, Acces B12 assay, Beckman Coulter, Brea, CA, USA). The blood cobalamin content was determined without previous sample preparation and with the competitive immunoassay method, as reported previously.

Plasmatic holotranscobalamin was determined by an immunoassay with an Active-B12 (Holotranscobalamin) ELISA kit (Ref. AX53101, IBL-International Gmbh, Hamburg, Germany), which was performed by following the manufacturer’s instructions. The reported range of detection was from 10 to 128 pmol/L.

The plasmatic homocysteine was determined by an enzymatic assay kit (Ref. 41057, Spinreact, Barcelona Spain), following the manufacturer’s instructions.

Methylmalonic acid was determined directly in the diluted urine samples by UPLC-MS/MS, following the procedure described by Boutin et al., (2020) [42]. In addition, a good chromatographic separation was obtained between the succinic and methylmalonic acids.

Ascorbic acid was determined by HPLC-DAD following the procedure described by Romeu-Nadal et al., (2006) [43]. Briefly, milk samples were thawed to around 22 °C in a water bath, protected from light, and then mixed. Dehydroascorbic acid was reduced to ascorbic acid with DL-dithiothreitol. Exactly 300 μL of human milk and 800 μL of DL-dithiothreitol 100 mM were added. The mixture was then shaken mechanically for 30 s and the tube was then kept in a dark place for 15 min. Next, 300 μL of meta-phosphoric acid (0.56% w/v) was added, the mixture was further shaken for 30 s, and then it was filtered with centrifuge filters at 4 °C for 30 min (Spin-X Micro Centrifuge Filter, 0.2 μm Nylon Filter). To analyze the ascorbic acid, 300 μL of breastmilk and 300 μL of meta-phosphoric acid (0.56% w/v) were mixed and filtered with centrifuge filters at 4 °C for 30 min. For chromatographic analysis, 50 μL of the filtrate was injected into an HPLC system. Isocratic chromatographic separation was carried out using a mobile phase of Milli-Q water with acetic acid (0.1% *v*/*v*) and methanol in a relative proportion of 95:5 (*v*/*v*). The effluent flowrate was 0.7 mL/min, and the column temperature was 25 °C. The analytical column used was a 5 μm Kromasil 100 Å, C18 (Tecknokroma, Barcelona, Spain). Ascorbic acid was identified by comparing the retention time of the sample peak with that of the ascorbic standard at 254 nm. Plasma samples were determined with the same methodology, with some modifications, as described by Robitaille and Hoffer (2016) [44], which were achieved mainly through the protein precipitation performed via 10% meta-phosphoric acid in 2 mM of EDTA.

The rest of the analysis for the water-soluble vitamins in human milk was carried out by UPLC tandem mass spectrometry (UPLC–MS/MS), following the method described by Hampel et al., (2012) [45]. Samples were subjected to protein precipitation and the removal of non-polar constituents by diethyl ether, prior to analysis. Quantification was performed via a ratio response to the stable-isotope-labeled internal standards. The limit of quantitation was between 0.05 and 5 ppb, depending on the vitamin. The water-soluble vitamins in the plasma and erythrocytes were determined with the same protocol that was used for its determination in human milk, with some modifications that were introduced in the sample preparation. Briefly, for 500 µL of the sample, protein precipitation was performed with the addition of 75 μL of trichloroacetic acid (500 g/L). Samples were vigorously mixed and then centrifuged at 10,500× *g* at 4 °C for 5 min. The supernatant was collected and mixed with 1 mL of methanol. Further samples were centrifuged at 10,500× *g* at 4 °C for 5 min. Moreover, the supernatant was dried under nitrogen and then finally resuspended with 500 µL of water.

The glutathione reductase activity in erythrocytes was determined by an Abcam ab83461 assay kit (Abcam, Cambridge, UK), following the manufacturer’s instructions.

#### 2.7.3. Fat-Soluble Vitamins

The concentrations of retinol (i.e., the main form of vitamin A in milk), α-tocopherol (the main biological form of vitamin E), and γ-tocopherol in the milk and plasma were determined by HPLC with a fluorescence and UV detector, following the method described by Jiang et al., (2016) [46]. The concentration of tocopherols was determined with an excitation wavelength of 295 nm and a cut-off emission filter of 345 nm. The retinol was determined by UV detection (325 nm wavelength). The external quantification was performed based on the calibration curves for retinol and α-tocopherol prepared each day of the analysis. Briefly, samples were saponificated with a mixed solution of 0.1 g ascorbic acid, 2 mL ethanol, including 0.1% BHT, and a 0.5 mL 50% aqueous potassium hydroxide solution. Further, the retinol and tocopherols were extracted with petroleum ether and the organic fraction was dried with nitrogen and then later re-dissolved with a mixed solution of methanol and methyl-tert-butyl-ether (1:1), including 0.1% BHT.

Vitamin D metabolites in milk and plasma were determined by UPLC electrospray ionization/tandem MS, as described by Aronov et al., (2008) [47]. Briefly, the samples’ protein precipitation was performed with acetonitrile. The vitamin D metabolites were extracted with methyl-tert-butyl-ether and the organic fraction was dried under nitrogen. Further, the samples were re-dissolved with methanol and 4-phenyl-1,2,4-triazoline-3,5-dione (PTAD) for its derivatization. The internal standards of the deuterated metabolites of vitamin D were used during the whole process.

### 2.8. Blood Biochemistry, Hemoglobin, and Urine Creatinine

Blood biochemistry was determined by the NUTREN-Nutrigenomics Group via enzymatic kinetic colorimetric methods. The total cholesterol, HDL- and LDL-cholesterol, and triacylglycerols were determined via enzymatic assays, following the manufacturer’s instructions (Refs. 1001090, 1001096, 41023, and 1001310, respectively; Spinreact, Barcelona, Spain). Hemoglobin was determined by the Drabkin colorimetric method, following the manufacturer’s instructions from the commercial assay kit (Ref. 1001230, Spinreact, Barcelona, Spain). The urine creatinine was determined by the Jaffé colorimetric kinetic method with a commercial assay kit (Ref. 1001110, Spinreact, Barcelona, Spain).

### 2.9. Human Milk Macronutrients Analysis

The breast milk macronutrient analyses were carried out at the RHMB. The leftover milk samples from the lipid study at the CIAL were received frozen. The milk was then thawed in a water bath and homogenized before analyses. Total fat, protein, and lactose in human milk samples were measured by Fourier transform mid-infrared (FT-MID) spectroscopy in a milk analyzer (MilkoScan FT2, FOSS S.A., Barcelona, Spain) properly calibrated for the analysis of human milk.

### 2.10. Statistics

The distribution of data for normality was evaluated using the Shapiro–Wilk test.

Quantitative sociodemographic variables were presented as the mean and standard deviation (SD) when they followed a parametric (normal) distribution and as median and interquartile range (IQR) when they followed a nonparametric distribution. Qualitative variables were expressed as both absolute and relative frequencies.

The lipid, vitamin, and mineral values were expressed as the mean and standard error (SE). In the case of the vitamins and minerals in the milk, due to the wide dispersion of the values of some of the nutrients, the median and IQR were presented. For the same reason, the median and IQR for the riboflavin intake were also shown.

The inferences on the qualitative variables were analyzed using a Chi-square test. The inferences on the quantitative variables were made depending on the distribution that was obtained via the use of the Student’s *t*-test or the Mann–Whitney U test for comparisons between two groups. A *p*-value < 0.05 was considered statistically significant. All statistical analyses were performed using SAS© software (SAS Institute Inc., Cary, NC, USA), version 9.4 of the SAS System for Windows.

## 3. Results

### 3.1. Population Studied

**Figure 2:**
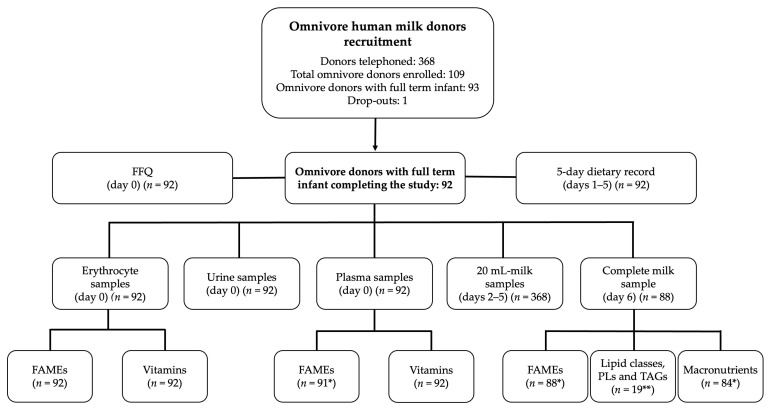
Flow chart for omnivore human milk donors’ recruitment and sampling. * The difference between 92 and the number of samples reflects missing data. ** Corresponding to a randomly selected subgroup of 19 omnivore donors with full-term infants. Abbreviations: FFQ, food frequency questionnaire; FAMEs, fatty acid methyl esters; PLs, relative composition of phospholipids; TAGs, molecular species of triacylglycerols.

**Figure 3:**
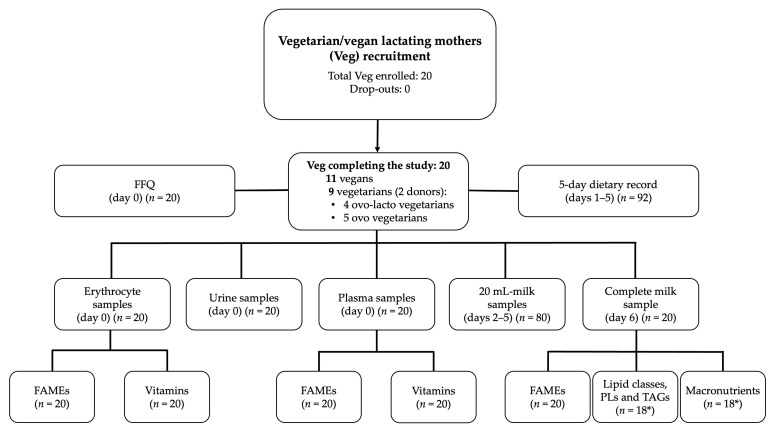
Flow chart for vegetarian/vegan lactating mothers’ (Veg) recruitment and sampling. * Due to two missing data. Abbreviations: FFQ, food frequency questionnaire; FAMEs, fatty acid methyl esters; PLs, relative composition of phospholipids; TAGs, molecular species of triacylglycerols.

A total of 113 women were recruited: 93 were Donors and 20 Veg. A total of 112 participants completed the study: 92 Donors and 20 Veg. In the Veg group, there were 11 vegans and 9 vegetarians (5 ovo-vegetarians and 4 ovo-lacto-vegetarians). Of the 20 Veg, 10 had made some change in their diet in the last 2 years. In particular, four had switched from vegetarian to vegan, two had switched from vegan to vegetarian, and one had occasionally consumed eggs, fish, and dairy during their pregnancy. Two of the vegetarians were human milk donors. The flow chart of subject recruitment and sampling is shown in Figure 2 and Figure 3.

Table 1, Table 2 and Table 3 show the comparison of health and socio-demographic data survey results between both groups. The only differences found were a lower gestational weight gain in the Veg group by a median of 1 kg (*p* value = 0.024), a higher percentage of women using manual milk expression in the Veg group (25% vs. 7.6%, *p* value = 0.038), and a different distribution of weight percentiles for the breastfed infants at birth (*p* value = 0.007) and at the time of the study (*p* value = 0.049). There was no difference in pre-pregnancy or in current BMI. The age range of breastfed infants was 2–50 months in the Donor group and 1–36 months in the Veg group.

The data reported by the participants on their consumption of pharmacological supplements during pregnancy and lactation are shown in Table 4. Most participants in both groups took supplements during gestation of vitamin B12, folic acid, and iodine. At the time of the study, a total of 17/20 (85%) Veg were taking B12 supplements, of which two were taking multi-nutrient supplements containing low doses of B12 (2.5 and 4 mcg/day), and 15 were taking high doses of B12 (1000–6000 mcg weekly). During lactation, there was a higher percentage of supplementation of vitamins A, E, C, B9, iodine, and iron in the Donors group. On the other hand, at the time of the study, higher supplementation doses of vitamins D, B7, B9, B12, and calcium were observed in the Veg group. The use of omega-3 supplements during pregnancy and lactation was low in the Veg group (30 and 35%, respectively).

### 3.2. Diet Survey

Table 5, Table 6 and Table 7 show the results of the five-day dietary records regarding the participants’ average daily nutrient intake together with recommended daily intakes (Table 5), the prevalence of inadequate intakes of specific nutrients (Table 6), and, furthermore, the number of food servings per day, the healthy eating index (HEI), and records of supplement and iodized salt intake (Table 7). Table 8 shows the results of the FFQ.

The caloric and lipid profiles of the diet of the Veg group showed to be more adequate. They consumed less saturated fat, *trans* FAs, and cholesterol, as well as more polyunsaturated fatty acids (PUFAs) consisting of linoleic acid (LA) and linolenic acid (ALA). However, their intake of very-long-chain *n*-3 FAs, DHA, and eicosapentaenoic acid (EPA) was found to be in a much lower than the recommended amount, as per the FAO/WHO (2008) [32]. Furthermore, there was a higher *n*-6/*n*-3 ratio in the Veg group. Regarding vitamins and minerals, the Donors consumed more riboflavin, niacin, calcium, phosphorus, and selenium, while Veg consumed more vitamin E, food folate + folic acid, and pyridoxine/proteins. The food folate and folic acid intake by the Donors was below the recommended amount (Table 5). However, there was no difference in the healthy eating index score between the two groups; both scores were indicative of a good diet (Table 7).

### 3.3. Nutritional Status

The results of the lipid, vitamin, and mineral studies in the erythrocytes, plasma, and urine are presented in Table 9 and Table 10. The contribution of the SFAs, monounsaturated fatty acids (MUFAs), and PUFAs to the total FAs content was similar in both the Donors and Veg group, with some small differences. The predominant FAs in both groups were the same: arachidonic acid (AA) followed by palmitic/stearic acids in the erythrocytes, and LA followed by palmitic/oleic acids in the plasma. Notably, Veg group had a lower proportion of SFAs in their plasma, but a significantly lower proportion of total *n*-3 FAs, and particularly of EPA and DHA, in both their plasma and erythrocytes, as well as a higher *n*-6/*n*-3 ratio. The levels of C16:0 DMA and C18:0 DMA and therefore the total content of plasmalogens were higher both in the plasma and erythrocytes from Donors (Table 9).

In the assessment of the participants’ riboflavin status from the measurement of the erythrocyte glutathione reductase activity coefficient (EGRAC), a high percentage of women with a deficiency was observed in both groups (29.7% in Donors and 57.1% in Veg). Vegetarians had lower plasma levels of riboflavin, higher levels of both erythrocyte and plasma pyridoxamine, higher plasma ascorbic acid, and lower urine calcium/creatinine ratios. The low levels of plasma thiamine, erythrocyte riboflavin (albeit normal–high plasma levels), plasma niacin, plasma folate, plasma 25-OHD_3_, and plasma α-tocopherol were noteworthy in both groups. On the other hand, the levels of plasma ascorbic acid, retinol, cobalamin, and holotranscobalamin II were normal in both groups (Table 10).

### 3.4. Human Milk Composition

The results of the macronutrient, lipid, vitamin, and mineral studies in human milk are presented in Table 11, Table 12 and Table 13.

There were no differences in the distribution of macronutrients and lipid classes in the milk of the two groups, except for a slight increase in the proportion of polar lipids in the milk of the Veg group (Table 11). The TAGs profile followed a similar distribution in the milk of both groups, with a clear predominance of long-chain molecular species in the Veg group. The content of the molecular species CN54 was significantly higher in the case of milk from the Veg mothers, which correlates with the higher proportion of oleic acid and LA acids that were present in that sample. Regarding the relative proportion of the different PLs in the milk, the majority compound was sphingomyelin (SM) in both groups, which accounted for more than 40% of the total polar lipids. However, in the Veg group, there was a higher proportion of phosphatidylethanolamine (PE) and a lower proportion of phosphatidylcholine (PC) than was found in the Donor group. In the FAs distribution (Table 12), the MUFAs were predominant in both groups. In comparison, the Veg group’s milk had lower relative levels of SFAs and higher relative levels of MUFAs and PUFAs than those in the milk of the Donors group. The predominant SFA was found to be palmitic acid in both groups, followed by myristic, lauric, and stearic acids. The predominant MUFA was oleic acid, the most abundant FA in the milk, and the predominant PUFA was LA in both groups. The Veg group had a higher proportion of LA and ALA and lower proportions of AA, DHA, and docosapentaenoic acid (DPA) in their milk. EPA was undetectable in all the samples. The DHA content in the Donors group was double that of the Veg group. Notably, the proportion of *n*-6 PUFAs in milk was higher in the Veg group, as was the ratio of *n*-6 PUFAs/*n*-3 PUFAs. Long-chain FAs containing more than 15 carbons were the predominant FAs in both diet groups, and there was no difference in the distribution of FAs according to chain length.

In the study of the micronutrient content of human milk (Table 13), the great variability found within was striking. Furthermore, this variability was much more evident in the case of riboflavin and retinol. For this reason, we have provided the data for both, the mean (standard error) and the median (interquartile range) for a proper interpretation of the data and to allow comparisons with other published studies. It should be noted that the cobalamin levels in milk were adequate in both groups, albeit higher in the Veg group. Adequate levels of vitamin C, α-tocopherol, pantothenic acid, iodine, and phosphorous were also found in the milk of both groups, but with higher levels of pantothenic acid and lower levels of phosphorous and iodine in the Veg group, and no differences between groups in the other two micronutrients. The vitamin D3 content in the milk was lower in the Veg group, but it was within the published range. The milk of the Veg group had a higher content of gamma-tocopherol than the Donors’ milk. On the other hand, the folate, nicotinamide, selenium, and calcium contents in the milk were low in both groups; all of them, except calcium, were lower in the Veg group. Nicotinamide showed the greatest difference between the two groups, as it was almost half in the milk of the Veg group compared to that of the Donors.

## 4. Discussion

The present study compares the maternal intake, nutritional status, and nutritional composition of human milk from 92 omnivore milk donors with full-term infants with that of 20 vegetarian/vegan lactating mothers in inland Spain.

We determined a large number of nutrients in the human milk, comprising macronutrients, lipid classes, sixteen molecular species of TAGs, three PLs, thirty FAs, eleven vitamins, and four minerals. There are no previous studies on vegetarians/vegans in which such a quantity of nutrients has been determined in their milk or in which they have been compared with milk donors. The previously measured compounds in vegetarians’ and in vegans’ milk have been limited to macronutrients, free amino acids, FAs, minerals, trace elements, taurine, glutathione peroxidase activity, nucleotides, nucleosides, brain-derived neurotrophic factor, folic acid, and vitamin B12. It is notable that the rest of the vitamins have not yet been studied. In addition, the literature lacks a more comprehensive nutritional and dietary study, as physical measurements were the only indicators in these studies on nutritional status [12].

In our study, the most important differences in the milk of the two dietary groups were found in the distribution of PLs and the FAs’ profile, which is in accordance with a notable difference in the type and amount of fat in the vegetarian diet that has been previously described [106] and which our study corroborates. On one hand, the Veg group consumed lower total fat, one-third less saturated fat, and markedly lower amounts of cholesterol and *trans* FAs. According to this, the milk of the Veg group was richer in unsaturated FAs and lower in SFAs and *trans* FAs, which is explained by the fact that plant-based diets are rich in unsaturated FAs. Veg showed a more favorable lipid profile, and their HDL plasma levels were higher. However, this group exhibited a plasmalogens content, both in plasma and erythrocytes, significantly lower than that of Donors. Plasmalogens have been proposed to act as reservoirs of PUFAs and, based on their ability to scavenge reactive oxygen species as well as to chelate potentially harmful metal ions, they are considered natural antioxidants [107]. So, these decreased levels in the Veg group might be a marker of a certain oxidative stress. Furthermore, with respect to essential FAs, the Veg group showed significant drawbacks, due to deficient intakes of DHA and EPA, as well as a low *n*-3 FAs intake relative to *n*-6 FAs intake. Relevant to this, the DHA intake by the Veg group (110 mg/day) was less than one-half of the DHA intake by the Donors (380 mg/day), which resulted in a DHA concentration in the milk of the Veg group that was less than half that of the Donors (0.15% vs. 0.33%, respectively). This is in line with the findings of another study comparing milk DHA content from non-donor vegetarians, vegans, and omnivores [108]. It is recommended that breastfeeding women consume at least 200 mg of DHA per day [109,110,111], which should translate into a milk DHA content of 0.30% [109]. The Veg group did not reach this recommendation. DHA supply to the fetus and neonate is associated with beneficial effects on visual function and cognitive development [109,110]. The impact of a low milk DHA content is greater for premature infants, as their DHA requirements are higher than those of term infants due to the lack of placental DHA transfer in the last trimester and the very low stores in adipose tissue [112]. In addition, they must ingest preformed DHA, as their ability to obtain DHA from ALA is thought to be very limited due to the decreased activity of their enzyme systems [113]. There are greater apparent benefits provided by a higher DHA concentration in milk for preterm infants (1 vs. 0.35% of the total FAs intake) [109,114], and DHA requirements for very-low-birth-weight infants have been established in the range from 12 to 60 mg/kg/day [109,115]. Considering that the pasteurization of DHM does not affect DHA levels [116,117], the DHA content in the milk of the Donors group was sufficient (albeit at the lower end of the adequate range) and that of the Veg group was clearly insufficient to meet the DHA needs of very-low-birth-weight infants. Calculations have been formulated, taking into account the lipid concentration found in the studied milk (3.13 g/dL), as well as the 0.33% vs. 0.15% DHA contents and enteral intake of 175 mL/kg/day.

However, we do not consider this to be a reason for the exclusion of vegetarian/vegan women as milk donors. Firstly, the intakes and levels of DHA in human milk vary widely across countries. Indeed, values equal or even lower than those of our Veg group have been reported both in developed and developing countries [118,119,120,121,122]. In fact, several studies on DHM in North America found DHA values that were similar to or even lower than those of our Veg group [116,117,123]. Secondly, since DHA supplementation to lactating women has consistently been shown to increase DHA concentrations in breast milk [124,125,126,127,128,129,130], it seems an appropriate strategy to recommend DHA supplementation from algal oil to Veg women who want to become milk donors. Most studies show that vegetarians, and especially vegans, consume low to zero amounts of EPA and DHA unless they take supplements [131]. This is because good sources of EPA and DHA are typically of animal origin, mainly cold-water fish and seafood. DHA-enriched eggs and milk are an option for ovo-lacto-vegetarians, but not for vegans. Some cold-water marine algae (other than spirulina) contain long-chain *n*-3 fatty acids, but their high iodine content limits their regular consumption. In addition, intakes of supplements up to 1 g/day DHA have been used in clinical trials with no significant adverse effects [110]. As only 25% of women in the Veg group reported taking DHA supplements, there is an important opportunity for improvement in this area.

DHA supplementation would even be a strategy to consider in the Donors group, especially in women with a low fish intake. A study conducted in the Mother’s Milk Bank of Ohio, in which the studied omnivores donors reported low basal dietary intakes of DHA (23 mg/day), concluded that supplementation with an algal DHA product at 1 g/day improved the dietary DHA and maternal milk concentration to mimic intrauterine accretion for the preterm infant [123]. Perrin found that the use of a DHA/EPA supplement by breastfeeding women was a significant positive predictor of milk ALA, DHA, and total *n*-3 composition, as well as a significant negative predictor of the *n*-6/*n*-3 ratio in milk [121].

The *n*-6/*n*-3 ratio of the diet, which is used to assess the balance between essential FAs and is recommended to be less than 10:1, is another issue of great interest. In the vegetarian and vegan populations, it should be set at 2:1–4:1, because they depend on the endogenous production of DHA and EPA from ALA, and higher *n*-6/*n*-3 ratios decrease it [106,131]. In the present study, the *n*-6/*n*-3 ratio was higher in the diet of the Veg group (9.9 vs. 7.8), and also in their erythrocytes, plasma, and milk. This is due to the high LA contents of plant-based diets, making it very difficult for vegans/vegetarians to obtain ALA without also increasing the amount of LA in the diet, unless specific foods that are high in ALA are consumed, such as flaxseed/linseed oil, hemp seeds, chia, or oleate-rich, low-LA sunflower oils [131]. Increasing the consumption of these foods could be an additional recommendation for vegetarians/vegans who want to be milk donors. Nevertheless, the human conversion rate of ALA to EPA and DHA is low, so in periods of high demand, such as in lactation, experts particularly recommend considering DHA supplementation in vegans [131].

The FAs profile in breast milk may affect the status of the infant. It was observed that in the total erythrocyte lipids of vegan-breastfed infants, the LA was higher, and the DHA was lower when compared to those of omnivore-breastfed infants [108]. However, more studies are needed because of the small sample size of the study that reported these results. Providing DHA and AA supplements to breastfed and very preterm infants in the early neonatal period was associated in another study with improved recognition memory and higher problem-solving scores at 6 months [132].

Regarding the PLs content of human milk, even though they constitute only about 1% of the total lipid content of milk, dietary PLs play an important role in the digestion, absorption, and transport of TAGs [133], as well as for infants’ intestinal maturation and the establishment of the intestinal immune system [134]. SM, PE, and PC are the three major PLs that have been found in human milk in most studies [135]. In agreement with certain studies [136,137], we found SM to be the most abundant PL in human milk, accounting for more than 40% of the total polar lipids. SM is located on the outside of the lipid bilayer of cell membranes and is particularly abundant in the myelin sheaths surrounding the axons of neurons. However, there are large differences in the content and distribution of PLs between studies that have been conducted around the world [136,137,138,139,140]. Further, PE was determined to be the most prevalent PL in human milk in some of them [138,139]. We found that the Veg group showed a lower percentage of PC (26.88% vs. 30.55%) and a higher percentage of PE (30.72% vs. 24.62%) in their milk. SM and PC are good sources of choline, whose importance lies in its involvement in the structural integrity of cell membranes, as a precursor of acetylcholine and as a methyl donor for methionine synthesis [138]. The clinical impact of these findings for preterm infants is unknown. To our knowledge, this is the first study to determine the relative composition of PLs in the milk of vegetarian/vegan women. Our study suggests the potential influence of dietary pattern on the distribution of PLs in human milk in two otherwise comparable population groups, but more studies are needed to support our findings.

In terms of micronutrients, the greatest concern among vegetarians is the vitamin B12 content of their milk because vitamin B12 is scarce in plant foods, and its deficiency in the lactating mother can lead to irreversible cognitive impairment and other neurological disorders as well as hematological complications in the breastfed infant [141]. This is a main reason for the exclusion of vegan women from milk donation in certain countries. In this respect, the vitamin B12 content in the milk of our Veg group is a perfect example that the vegetarian/vegan diet can be suitable for lactating women if sufficient supplementation is provided. Our results support the approach of most worldwide milk banks, which recruit vegetarian/vegan mothers as donors on the condition that they regularly supplement vitamin B12, as well as other micronutrients if necessary [12]. In the Veg group, with a vitamin B12 supplementation rate of 85% and an average daily dose of 312 mcg/day, their plasmatic levels were similar to those of the Donors. In addition, the vitamin B12 content of milk was found to be higher than that of the Donors, and the content of cobalamin in the milk of both groups was found to be adequate. A total of 14/20 (70%) of the Veg consumed supplements with high doses of B12 that were equal to or higher than the 2000 mcg weekly amount recommended by the American Dietetic Association and other authors [142,143].

Of the remaining vitamins and minerals, the differences found in the content of certain vitamins in the milk between the Veg and Donors were probably of little clinical relevance and would not justify the exclusion of the vegetarian/vegan group studied from milk donation. Nicotinamide showed the greatest difference between the two groups, as it was almost half in the Veg group compared to the Donors. Interestingly, we found that even though the Veg group consumed more vitamin E, they did not have higher plasma or milk concentrations of α-tocopherol. However, they did have higher plasma and milk levels of γ-tocopherol, which is a vitamer of growing interest for its detoxifying, anti-inflammatory, natriuretic, and protective properties against cardiovascular disease and prostate cancer [81,144]. In general, the results of the milks’ vitamin analysis appeared to be in line with the results of the dietary record analysis, except in the case of folate and vitamin D. It is known that the folate content in milk does not depend on the mother’s intake [145], and the vitamin D status depends not only on her intake, but also on sun exposure. Except for an old study in which no significant differences in the milk’s folate content were found between lacto-vegetarians and omnivores [146], there have been no studies evaluating other vitamins (except cobalamin) in the milk of vegetarians/vegans. Additionally, there are few studies assessing the concentration of vitamins in raw DHM [5,147]. Studies assessing the mineral content in both vegetarians/vegans and milk donors are also scarce [5,12,15]. We have, therefore, compared our values in the milk with those used by the European Food Safety Authority (EFSA) [91,92,93,94,95,96,97,98,99,102,103,104] in order to establish the micronutrient adequate intakes for infants under 6 months of age. In cases in which we were not able to measure the total content of a vitamin in milk, because we determined only some of its vitamers (i.e., free thiamine, free riboflavin, nicotinamide, and pyridoxal), we compared our vitamers’ values with those of Gibson’s study [90]. The low content of calcium, folate, nicotinamide, and selenium in both the Veg and Donors groups was remarkable. In this regard, plasmatic nicotinamide was low in both groups. In addition, the folate stores were deficient in a high percentage of lactating mothers in both the Veg and Donors groups. The high percentage of women in both groups who showed biochemical values suggestive of low stores of some vitamins (B1, B2, B3, B9, and vitamins D and E) was alarming, especially regarding vitamin E; although it must be said that the values of α-tocopherol in milk were adequate. These values are difficult to interpret, mainly because there are nearly no studies assessing the biochemical nutritional status of lactating women in developed countries. As the Institute of Medicine (IOM) stated in 1991 in its document “Nutrition during lactation”, “no standards for anthropometric or biochemical indicators have been established for nutritional status among lactating women” and “it is probably inappropriate to assess the nutritional status of lactating women by comparing plasma nutrient values with reference values for a nonpregnant women” [148]. Moreover, differences in analytical techniques between studies make it even more difficult to interpret results. The incorporation of modern techniques in our study based on liquid chromatography and tandem mass spectrometry allowed us to characterize more specifically the different group B vitamers, but for some of them, there are still no established and universally accepted reference values. Another unexplored factor is how the duration of breastfeeding affects the mother’s nutrient stores. The average breastfeeding time of the women in our study was 7–8 months, whereby more than half of them were breastfeeding for more than 6 months, and some of them for up to 50 months, with 5% of them conducting tandem breastfeeding. It should be remembered that the milk donors not only breastfeed their own children, but also express milk for donation. All of this could affect their nutrient stores. We found a study conducted by Papathakis et al. in 2007 [80], which showed that 70% of lactating South African women at 24 weeks postpartum met the criteria for vitamin E deficiency (< 11.6 mcM) and the mean plasmatic α-tocopherol decreased significantly over time after their delivery. In Gibson’s study [90], in Indonesian disadvantaged mothers, most micronutrient concentrations in milk declined during lactation, independent of changes in human milk production. Furthermore, there were significant associations between maternal biomarkers and milk micronutrient concentrations at 5 months, thereby raising the need for further studies to investigate a possible link between the decline in milk micronutrients and the shifts in maternal status. Clearly, more studies are needed to assess the biochemical parameters of the nutritional status of lactating women to help establish the ranges of normality and deficiency in this population. In addition, studies that assess the behavior of maternal nutrient stores in the face of prolonged breastfeeding are required.

Regarding the mineral content of milk, we found statistically significant differences in three of the four minerals studied between the two investigated groups. Iodine, phosphorus, and selenium were lower in the milk of the Veg group. These results contrast with those of Perrin [15] and Debski [149], who found higher selenium content in the milk of women with a plant-based diet when compared to omnivore women. In our case, women in the Veg group consumed less selenium and phosphorus. The milk phosphorus content was within the reference range in both groups. However, selenium levels were low in both groups—15% lower in the Veg group compared to that of the Donors. Although there was no difference in iodine intake, the median urine iodine concentration showed that the Veg group is an iodine-deficient population, while the Donors group are iodine-sufficient. The milk iodine concentration was within the normal range in both groups, but was 20% lower in the Veg group, which may be of relevance in meeting the high requirements of preterm infants [150]. More clinically important was the low calcium content in the milk of both groups, which may mean that the Spanish women’s calcium requirements are higher than those dictated by the EFSA and IOM, according to the recommended calcium intake of 1500 mg/day for Spanish lactating women [24], which neither group reached.

Another point worth mentioning is that a lower gestational weight gain in the Veg group and lower weight percentiles of their offspring both at birth and at the time of the study were found. In this regard, vegetarian diets were associated with lower gestational weight gain in a systematic review of observational studies [151]. Furthermore, in a review on the effects of vegetarian and vegan diets during pregnancy on the health of mothers and their offspring, several studies reported an association between a vegetarian diet and lower fetal growth, lower birth weight, or a higher risk of being small for their gestational age [152].

Our study presents certain limitations. It is known that the dietary patterns and levels of the biochemical indicators regarding nutritional status can be quite different between vegetarians and vegans [121,131], but we were not able to differentiate between the vegetarian and vegan mothers because of the low sample sizes. However, this study is still one of the most complete investigations available as it not only studies the nutritional content of the milk, but also is supported by a detailed dietary study that includes a five-day dietary record, as well as biochemical determinations of the plasma of lactating women. Another strength is the multiple milk samples that were collected from each woman over four consecutive days.

Finally, we would like to emphasize that the studied milk was mature milk, which was subjected to the usual procedures in the donation process, such as extraction, freezing, and thawing. Similarly, storage was achieved by utilizing transparent glass bottles to mimic the usual donation procedure, whereby the photosensitive vitamins were not protected from light. All these processes can affect the composition of the milk to a greater or lesser extent [153,154,155]. Therefore, the results are not superimposable to those of mothers’ own milk, but they do reflect the nutritional content of raw DHM. The effect of pasteurization on milk micronutrients has also been studied previously [156,157].

## 5. Conclusions

In conclusion, perhaps the most important contribution of this study is the detailed and comprehensive description of micronutrients and lipids in human milk from omnivore milk donors and vegetarian/vegan women and their comparison with the reference values. The milk of vegetarians and vegans has been shown to be different from that of omnivore donors, probably according to the differences in their dietary pattern, thereby showing advantages and disadvantages that we should be aware of. Of particular concern is the lower DHA content in the milk of our vegetarians/vegans’ group. However, raising awareness and administering proper supplementation could bridge this gap, as has been the case with vitamin B12.

In view of the results of this study, in the donor selection processes going forward, it might be more appropriate to conduct an individualized dietary assessment than to simply accept or reject a woman as a potential donor depending on whether she is omnivorous or vegetarian/vegan. An additional consideration is that, due to the high variability found in the micronutrient content and lipid profile in human milk, it is recommended for milk bank pooling practices to decrease the compositional variability in DHM [116,158].

## Figures and Tables

**Table 1:** Characteristics of the omnivore human milk donors with full-term infants (Donors) and lactating vegetarian/vegan mothers (Veg).

Characteristic	Donors (*n* = 92)	Veg (*n* = 20)	*p* Value
Age (years)	36.1 (4.2); 28–47	33.9 (5,2); 24–42	0.118
Weight (kg)	60.8 (55.3, 71.0); 46–122	61.1 (52.8, 64.9); 48–93	0.365
Height (cm)	164.2 (6.3); 152–158	163.1 (4.4); 155–172	0.493
Pre-pregnancy BMI (kg/m^2^)	22.3 (21.0, 25.0); 18.2–44.2	22.2 (20.1, 23.9); 18.0–34.3	0.436
Pre-pregnancy BMI (kg/m^2^) category			
Underweight (<18.5)	1 (1.1%)	2 (10.0%)	0.135
Normal (18.5–24.9)	69 (75.0%)	15 (75.0%)	
Overweight (25–29.9)	12 (13.0%)	2 (10.0%)	
Obese (≥30)	10 (10.9%)	1 (5.0%)	
Current BMI (kg/m^2^)	23.1 (21.2, 25.0); 16.7–42.8	22.8 (20.5, 23.7); 18.3–38.9	0.287
Current BMI (kg/m^2^) category			
Underweight (<18.5)	2 (2.2%)	1 (5.0%)	0.624
Normal (18.5–24.9)	67 (72.8%)	16 (80.0%)	
Overweight (25–29.9)	12 (13.0%)	1 (5.0%)	
Obese (≥30)	11 (12.0%)	2 (10.0%)	
Gestational weight gain (kg)	12.0 (9.2, 15.0); 5–30	11.0 (8.5, 12.2); 6–13.6	0.024
Postpartum weight retention (kg)	0.75 (−0.8–2.2); −12–11.8	0.7 (−1.8–3.2); −4.2–11.2	0.846
Number of children			
1	48 (52.2%)	14 (70.0%)	0.557
2	36 (39.1%)	6 (30.0%)	
≥3	8 (8.7%)	0 (0.0%)	
Country of origin: Spain, *n* (%)	84 (91.3%)	19 (95.0%)	0.581
Education level			
Secondary studies	2 (2.2%)	3 (15.0%)	0.063
Technical studies	9 (9.8%)	1 (5.0%)	
University studies	81 (88.0%)	16 (80.0%)	
Currently working	43 (46.7%)	14 (70.0%)	0.059
Physical activity			
Sedentary	21 (22.8%)	5 (25.0%)	0.355
Low active	49 (53.3%)	7 (35.0%)	
Active/very active	22 (23.9%)	8 (40.0%)	
Tobacco consumption			
Previously	18 (19.6%)	4 (20.0%)	0.96
Currently			
Passive smoking	18 (19.6%)	2 (10.0%)	0.16
Active smoking	1 (1.1%)	1 (5.0%)	
Alcohol consumption			
Prior to pregnancy	47 (51.1%)	11 (55.0%)	0.228
During pregnancy	1 (1.1%)	1 (5.0%)	
Currently	4 (4.3%)	3 (15.0%)	
Season during the study			
Spring	24 (26.1%)	1 (5.0%)	0.103
Summer	13 (14.1%)	6 (30.0%)	
Autumn	33 (35.9%)	8 (40.0%)	
Winter	22 (23.9%)	5 (25.0%)	

The quantitative sociodemographic variables are presented as means (standard deviations) when they followed a parametric distribution and as medians (25th, 75th percentiles) when they followed a nonparametric distribution. The ranges are displayed after the semicolons. The qualitative variables are expressed as the absolute and relative frequencies (%). Abbreviations: BMI, body mass index.

**Table 2:** Characteristics of the breastfed offspring of the omnivore human milk donors with full-term infants (Donors) and vegetarian/vegan lactating mothers (Veg).

Characteristic	Donors (*n* = 92)	Veg (*n* = 20)	*p* Value
Girl	50 (54.3%)	7 (35%)	0.116
Boy	42 (45.7%)	13 (65.0%)	
Twin pregnancy	0 (0.0%)	1 (5.0%)	0.178
Gestational age (weeks)	39^+6^ (39, 40^+4^); 37^+1^–42^+3^	39^+4^ (38^+6^, 40^+3^); 36^+4^–41^+4^	0.082
Birth weight (grams)	3303.4 (412.7); 2120–4640	3125.8 (412.6); 2240–3960	0.120
Birth weight percentile ^1^			
≤25	23 (25.0%)	12 (60.0%)	0.007
25–75	62 (67.4%)	8 (40.0%)	
≥75	7 (7.6%)	0 (0.0%)	
Age of breastfed child (months)			
0–6	34 (37.0%)	9 (45.0%)	0.776
6–12	34 (37.0%)	6 (30.0%)	
12–50	24 (26.1%)	5 (25.0%)	
Weight percentile of breastfed child ^2^			
≤15	13 (14.1%)	7 (35.0%)	0.049
15–85	62 (67.4%)	12 (60.0%)	
≥85	17 (18.5%)	1 (5.0%)	

The quantitative sociodemographic variables are presented as means (standard deviations) when they followed a parametric distribution and as medians (25th, 75th percentiles) when they followed a nonparametric distribution. The ranges are displayed after the semicolons. The qualitative variables are expressed as the absolute and relative frequencies (%). ^1^ Taking as reference the Olsen intrauterine growth curves [48]. ^2^ Taking as reference the World Health Organization (WHO)’s child growth standards [49].

**Table 3:** Characteristics of the lactation of the omnivore human milk donors with full-term infants (Donors) and vegetarian/vegan lactating mothers (Veg).

Characteristic	Donors (*n* = 92)	Veg (*n* = 20)	*p* Value
Donor previously	19 (20.6%)	1 (5%)	0.118
Lactation stage (months)	7.0 (5.0, 13.5); 2–50	8.0 [4.5–14.0]; 1–36	0.942
Type of lactation			
Exclusive	40 (43.5%)	9 (45%)	0.901
Partial	52 (56.5%)	11 (55%)	
Sum of child direct breastfeeding times plus daily pumped sessions			
<5	8 (8.7%)	2 (10.0%)	0.971
5–10	59 (64.1%)	12 (60.0%)	
>10	24 (26.1%)	5 (25.0%)	
Missing data	1 (1.1%)	1 (5.0%)	
Tandem breastfeeding	5 (5.4%)	1 (5.0%)	0.937
Breastfeeding twins	0 (0%)	0 (0%)	-
Type of milk expression *			
Manual	7 (7.6%)	5 (25%)	0.038
Mechanical breast pump	10 (10.9%)	2 (10.0%)	0.909
Simple electric breast pump	68 (73.9%)	14 (70%)	0.720
Double electric breast pump	12 (13.0%)	2 (10%)	0.709

The quantitative sociodemographic variables are presented as means (standard deviations) when they followed a parametric distribution and as medians (25th, 75th percentiles) when they followed a nonparametric distribution. The ranges are displayed after the semicolons. The qualitative variables are expressed as the absolute and relative frequencies (%). * The categories are not excluding each other.

**Table 4:** Consumption of the pharmacological supplements during pregnancy and the lactation of the omnivore human milk donors with full-term infants (Donors) and vegetarian/vegan lactating mothers (Veg).

Pharmacological Supplement	*n* (%)		*p* Value	Daily Dose, Mean (SE); Range		*p* Value
	Donors (*n* = 92)	Veg (*n* = 20)		Donors (*n* = 92)	Veg (*n* = 20)	
Vitamin A, mcg						
Pregnancy	12 (13.0)	3 (15.0)	0.732	527.6 (69.7); 23.0–700.0	383.3 (174.0); 100.0–700.0	0.389
Lactation	45 (48.9)	3 (15.0)	0.002			
Previously	14 (15.2)	2 (10.0)		730.9 (49.2); 333.0–1000.0	250.0 (150.0); 100.0–400.0	0.022
Currently	31 (33.7)	1 (5.0)		678.1 (45.2); 160.0–1000.0	800.0 (-); 800.0–800.0	0.729
Vitamin D, mcg						
Pregnancy	50 (54.3)	10 (50.0)	0.687	10.61 (0.8); 3.8–30.0	17.08 (6.2); 5.0–62.5	0.925
Lactation	52 (56.5)	9 (45.0)	0.220			
Previously	15 (16.3)	2 (10.0)		5.0 (0.4); 2.5–10.0	6.2 (3.7); 2.5–10.0	0.926
Currently	37 (40.2)	7 (35.0)		6.0 (0.8); 1.0–25.0	27.4 (9.0); 5.0–62.5	0.001
Vitamin E, mg						
Pregnancy	22 (23.9)	7 (35.0)	0.318	10.7 (0.7); 1.8–15.0	11. (2.0); 4.0–20.0	0.762
Lactation	49 (53.3)	6 (30.0)	0.030			
Previously	15 (16.3)	2 (10.0)		11.5 (0.5); 6.0–15.0	13.0 (7.0); 6.0–20.0	0.926
Currently	34 (37.0)	4 (20.0)		10.9 (0.6); 2.4–16.0	11.0 (0.6); 10.0–12.0	0.542
Vitamin C, mg						
Pregnancy	48 (52.2)	10 (50.0)	0.823	61.1 (4.2); 12.0–180.0	93.6 (30.6); 26.0–358.0	0.336
Lactation	51 (55.4)	7 (35.0)	0.050			
Previously	16 (17.4)	3 (15.0)		80.0 (4.2); 40.0–110.0	60.0 (20.0); 40.0–100.0	0.260
Currently	35 (38.0)	4 (20.0)		73.5 (4.2); 16.0–125.0	65.0 (8.7); 50.0–80.0	0.492
Vitamin B1, thiamine, mg						
Pregnancy	48 (52.2)	9 (45.0)	0.530	1.1 (0.0); 0.6–1.5	3.3 (1.7); 0.9–17.0	0.038
Lactation	50 (54.3)	8 (40.0)	0.149			
Previously	16 (17.4)	3 (15.0)		1.0 (0.1); 0.6–1.2	2.8 (1.1); 0.6–4.3	0.221
Currently	34 (37.0)	5 (25.0)		0.9 (0.1); 0.2–1.1	10.8 (9.8); 0.9–50.0	0.431
Vitamin B2, riboflavin, mg						
Pregnancy	48 (52.2)	9 (45.0)	0.530	1.4 (0.0); 0.7–2.5	2.4 (0.8); 1.0–8.5	0.060
Lactation	50 (54.3)	8 (40.0)	0.149			
Previously	16 (17.4)	3 (15.0)		1.3 (0.1); 0.7–1.6	2.9 (1.2); 0.7–5.0	0.272
Currently	34 (37.0)	5 (25.0)		1.3 (0.1); 0.7–1.6	5.0 (3.8); 1.0–20.0	0.750
Vitamin B3, niacin, mg						
Pregnancy	48 (52.2)	8 (40.0)	0.301	15.6 (0.4); 4.0–20.0	17.4 (2.6); 10.0–33.0	0.375
Lactation	50 (54.3)	8 (40.0)	0.149			
Previously	16 (17.4)	3 (15.0)		15.0 (0.7); 8.0–16.0	14.0 (3.1); 8.0–18.0	0.718
Currently	34 (37.0)	5 (25.0)		13.3 (0.7); 3.2–16.0	11.4 (2.1); 5.0–16.0	0.403
Vitamin B5, pantothenic, mg						
Pregnancy	48 (52.2)	8 (40.0)	0.301	5.7 (0.2); 4.0–20.0	8.9 (3.2); 5.0–31.0	0.923
Lactation	50 (54.3)	8 (40.0)	0.149			
Previously	16 (17.4)	3 (15.0)		5.6 (0.3); 3.0–6.0	6.3 (2.0); 3.0–10.0	0.718
Currently	34 (37.0)	5 (25.0)		5.1 (0.2); 2.8–6.0	24.4 (18.9); 5.0–100.0	0.488
Vitamin B6, pyridoxine, mg						
Pregnancy	48 (52.2)	9 (45.0)	0.530	1.4 (0.0); 0.7–2.2	3.6 (1.5); 0.8–2.5	0.533
Lactation	50 (54.3)	8 (40.0)	0.149			
Previously	16 (17.4)	3 (15.0)		1.4 (0.1); 0.7–2.2	3.0 (1.4); 0.7–5.4	0.249
Currently	34 (37.0)	5 (25.0)		1.3 (0.1); 0.3–2.0	3.6 (2.2); 1.3–12.5	0.617
Vitamin B7, biotin, mcg						
Pregnancy	48 (52.2)	8 (40.0)	0.301	55.3 (2.9); 25.0–150.0	117.1 (20.1); 50.0–187.0	<0.001
Lactation	50 (54.3)	7 (35.0)	0.063			
Previously	16 (17.4)	2 (10.0)		47.5 (2.3); 25.0–60.0	37.5 (12.5); 25.0–50−0	0.208
Currently	34 (37.0)	5 (25.0)		42.3 (2.1); 10.0–50.0	116.0 (27.5); 50.0–180.0	0.002
Vitamin B9, folic acid, mcg						
Pregnancy	89 (96.7)	20 (100.0)	0.999	603.6 (93.5); 162.0–6200.0	636.5 (229.9); 286.0–5000.0	0.990
Lactation	75 (81.5)	13 (65.0)	0.022			
Previously	19 (20.7)	6 (30.0)		272.4 (24.3); 100–400	416.7 (91.0); 100.0–800.0	0.094
Currently	56 (60.9)	7 (35.0)		280.1 (16.2); 2.0–400	400.0 (21.8); 300.0–500.0	0.011
Vitamin B12, cobalamin, mcg						
Pregnancy	88 (95.7)	20 (100.0)	0.999	2.3 (0.1); 0.95–4.7	213.5 (55.8); 1.2–934.0	0.002
Lactation	74 (80.4)	18 (90.0)	0.683			
Previously	19 (20.7)	1 (5.0)		2.4 (0.1); 1.3–3.5	2.0 (-); 2.0–2.0	0.213
Currently	55 (59.8)	17 (85.0)		2.1 (0.1); 1.0–2.5	312.1 (48.0); 2.0–857.0	<0.001
Iodine, mcg						
Pregnancy	89 (96.7)	19 (95.0)	0.452	199.0 (3.9); 46.5–400.0	177.7 (10.1); 75.0–229.4	0.076
Lactation	78 (84.8)	12 (60.0)	0.001			
Previously	18 (19.6)	5 (25.0)		191.7 (6.1); 100.0–200.0	185.8 (22.2); 100.0–229.0	0.693
Currently	60 (65.2)	7 (35.0)		183.2 (5.9); 46.0–300.0	157.1 (22.3); 75.0–200.0	0.101
Calcium, mg						
Pregnancy	3 (3.3)	4 (20.0)	0.019	62.0 (44.1); 12.0–150.0	391.8 (149.7); 100.0–650.0	0.075
Lactation	37 (40.2)	5 (25.0)	0.144			
Previously	12 (13.0)	2 (10.0)		180.8 (17.1); 24.0–245.0	225.0 (125.0); 100.0–350.0	0.749
Currently	25 (27.2)	3 (15.0)		164.0 (17.9); 40.0–500.0	566.7 (83.3); 400.0–650.0	0.004
Iron, mg						
Pregnancy	67 (72.8)	15 (75.0)	0.899	45.6 (3.6); 6.5–108.0	46.9 (6.0); 9.0–80.0	0.540
Lactation	66 (71.7)	10 (50.0)	0.021			
Previously	26 (28.3)	5 (25.0)		39.9 (6.3); 7.0–105.0	69.0 (7.0); 47.0–80.0	0.061
Currently	40 (43.5)	5 (25.0)		29.2 (5.3); 3.0–114.0	40.4 (16.2); 14.0–80.0	0.302
Zinc, mg						
Pregnancy	44 (47.8)	8 (40.0)	0.498	9.7 (0.2); 4.3–15.0	11.9 (2.1); 7.5–25.0	0.603
Lactation	48 (52.2)	7 (35.0)	0.097			
Previously	16 (17.4)	3 (15.0)		9.2 (0.5); 5.0–10.0	13.3 (6.0); 5.0–25.0	0.718
Currently	32 (34.8)	4 (20.0)		8.3 (0.5); 2.0–10.0	7.8 (1.0); 5.0–10.0	0.302
Selenium, mcg						
Pregnancy	42 (45.7)	7 (35.0)	0.363	52.3 (1.3); 27.0–60.0	40.0 (8.0); 12.5–60.0	0.173
Lactation	46 (50.0)	6 (30.0)	0.054			
Previously	15 (16.3)	2 (10.0)		26.8 (4.0); 10.0–55.0	32.5 (22.5); 10.0–55.0	0.937
Currently	31 (33.7)	4 (20.0)		27.1 (3.4); 4.0–55.0	34.0 (12.1); 13.0–55.0	0.579
Omega 3, g						
Pregnancy	48 (52.2)	6 (30.0%)	0.065	0.23 (0.02); 0.16–0.95	0.21 (0.04); 0.10–0.38	0.550
Lactation	48 (52.2)	7 (35.0%)	0.097			
DHA, g						
Lactation						
Previously	15 (16.3)	2 (10.0%)		0.18 (0.01); 0.08–0.20	0.10 (-); 0.10–0.10	0.102
Currently	33 (35.9)	5 (25.0%)		0.18 (0.01); 0.10–0.38	0.18 (0.03); 0.10–0.25	0.546
EPA, g						
Lactation						
Previously	15 (16.3)	2 (10.0)		0.03 (0.00); 0.00–0.04	0.02 (-); 0.02–0.02	0.332
Currently	33 (35.9)	5 (25.0)		0.05 (0.02); 0.00–0.50	0.15 (0.12); 0.00–0.64	0.727

The quantitative variables are presented as means (standard error of the mean). The ranges are displayed after the semicolons. The qualitative variables are expressed as the absolute and relative frequencies (%). Women who took supplements during lactation but had stopped taking them at the time of the study were classified in the “Previously” group. Women who were still taking supplements at the time of the study were classified in the “Currently” group. Abbreviations: SE, standard error of the mean; DHA, docosahexaenoic acid; and EPA, eicosapentaenoic acid.

**Table 5:** Diet survey: five-day dietary record. Daily nutrients intake of the omnivore human milk donors with full-term infants (Donors) and vegetarian/vegan lactating mothers (Veg), compared with recommended daily intakes.

	Donors (*n* = 92)	Veg (*n* = 20)	*p* Value	Recommendations ^a^	
				EFSA (PRI/AI *)	IOM (RDA/AI *)
Energy (Kcal)	2318.66 (43.49)	2146.73 (85.06)	0.079	^b^	
Protein (g)	96.36 (1.95)	67.46 (3.12)	<0.001	^c^	71
Total fat (g)	102.66 (2.62)	85.81 (4.55)	0.004		
Saturated fat (g)	33.12 (1.01)	19.88 (1.51)	<0.001	ALAP	ALAP
Polyunsaturated fat (g)	16.29 (0.58)	21.00 (1.42)	0.001		
Monounsaturated fat (g)	43.94 (1.21)	38.06 (2.48)	0.040		
PUFAs/SFAs	0.54 (0.02)	1.28 (0.11)	<0.001		
(PUFAs + MUFAs)/SFAs	1.94 (0.05)	3.54 (0.23)	<0.001		
Kcal from carbohydrate (%)	43.76 (0.60)	51.83 (0.92)	<0.001	45–60 **	45–65 **
Kcal from protein (%)	16.84 (0.26)	12.65 (0.45)	<0.001		10–35 **
Kcal from fat (%)	39.24 (0.60)	35.29 (0.95)	0.003	20–35 **	20–35 **
Kcal from saturated fat (%)	12.66 (0.23)	8.18 (0.54)	<0.001		
Kcal from polyunsaturated fat (%)	6.30 (0.19)	8.72 (0.35)	<0.001		
Kcal from monounsaturated fat (%)	16.81 (0.33)	15.72 (0.67)	0.239		
Kcal from *n*-3 fatty acids (%)	0.84 (0.03)	0.89 (0.10)	0.858	0.5	0.6–1.2
*n*-6 fatty acids (g)	13.64 (0.51)	18.27 (1.28)	<0.001		13 *
*n*-3 fatty acids (g)	2.11 (0.09)	2.37 (0.31)	0.846		1.3 *
*n*-6/*n*-3 fatty acids	7.77 (0.27)	9.90 (0.69)	0.002		
Myristic acid C14:0 (g)	2.86 (0.14)	1.28 (0.26)	<0.001		
Palmitic acid C16:0 (g)	16.52 (0.50)	8.40 (0.67)	<0.001		
Palmitoleic acid C16:1 n7 (g)	1.55 (0.06)	0.42 (0.06)	<0.001		
Stearic acid C18:0 (g)	7.07 (0.23)	3.53 (0.33)	<0.001		
Oleic acid C18:1n9c (g)	40.58 (1.16)	36.40 (2.35)	0.106		
Linoleic acid C18:2n6c (g)	13.45 (0.51)	18.25 (1.28)	<0.001		
Linolenic acid C18:3n3 (g)	1.58 (0.07)	2.24 (0.30)	0.016		
Eicosapentaenoic acid C20:5n3 (g)	0.15 (0.02)	0.04 (0.02)	<0.001		
Docosapentaenoic acid C22:5n3 (g)	0.08 (0.03)	0.00 (0.00)	<0.001		
Docosahexaenoic acid C22:6n3 (g)	0.38 (0.03)	0.11 (0.03)	<0.001	+0.10–0.20 *^d^	
EPA + DHA (g)	0.53 (0.04)	0.14 (0.04)	<0.001	0.25 *	
Trans fatty acids (g)	0.44 (0.02)	0.13 (0.03)	<0.001	ALAP	ALAP
Cholesterol (g)	334.42 (10.56)	58.60 (17.24)	<0.001		ALAP
Cholesterol (mg/1000 Kcal)	144.96 (4.03)	28.90 (8.45)	<0.001		
Thiamine (B_1_) (mg)	2.02 (0.07)	4.75 (2.46)	0.056	0.1 mg/MJ	1.4 mg
Riboflavin (B_2_) (mg)	2.55 (0.09) 2.40 (1.70, 3.20) ^1^	2.95 (0.98) 1.70 (1.20, 2.80) ^1^	0.024	2.0	1.6
Niacin (B_3_) (mg)	43.21 (1.09)	31.24 (1.75)	<0.001	1.6 mg/MJ ^e^	17 mg ^e^
Pantothenic acid (B_5_) (mg)	7.93 (0.31)	12.28 (4.94)	0.551	7 *	7 *
Pyridoxine (B_6_) (mg)	2.91 (0.10)	3.40 (0.60)	0.997	1.7	2
Biotin (B_7_) (μg)	51.60 (2.94)	63.86 (12.69)	0.779	45 *	35 *
Folate food + folic acid (B_9_) (μg)	473.22 (20.87)	668.15 (46.65)	<0.001	500 ^f^	500 ^g^
Cobalamin (B_12_) (μg)	6.92 (0.28)	258.40 (53.44)	0.096	5 *	2.8
Vitamin C (mg)	178.41 (8.87)	211.86 (18.33)	0.088	155	<19y: 115
					≥19y: 120
Vitamin A (μg)	1430.37 (111.15)	1357.94 (108.26)	0.704	1300 ^h^	<19y: 1200 ^i^
					≥19y: 1300 ^i^
Vitamin D (μg)	5.61 (0.47)	10.78 (4.28)	0.204	15 *^j^	15 ^jk^
Vitamin E (μg)	17.16 (0.84)	20.65 (1.49)	0.029	11 *^l^	19
Iodine (μg)	245.33 (11.76)	259.58 (47.15)	0.536	200 *	290
Calcium (mg)	1148.32 (35.12)	910.92 (70.48)	0.002	18–24y:1000	<19y: 1300
				≥25y: 950	≥19y: 1000
Phosphorus (mg)	1677.19 (38.38)	1436.13 (79.71)	0.007	550 *	<19y: 1250
					≥19y: 700
Iron (mg)	25.34 (2.17)	31.33 (5.23)	0.028	16	<19y: 10
					≥19y: 9
Zinc (mg)	14.40 (0.52)	12.07 (0.83)	0.077	10.4–15.6 ^m^	<19y: 13
					≥19y: 12
Selenium (μg)	118.98 (3.38)	100.80 (6.66)	0.010	85 *	70

The quantitative variables are presented as means (standard error of the mean). ^1^ Medians and interquartile ranges for riboflavin are also shown, due to the wide dispersion of the values in the Veg group. ^a^ Recommended daily intake. Adequate intake is presented with an asterisk (*) and PRI/RDA (i.e., Population Reference Intake for the EFSA values, and Recommended Dietary Allowance for the IOM values) in ordinary type. ** Reference intake range. ^b^ Depends on age and level of physical activity. ^c^ 0.83 g/kg body weight + 19 g/day from 0–6 months postpartum or + 13 g/day if >6 months postpartum. ^d^ In addition to the combined intakes of EPA and DHA of 0.25 g/day. ^e^ As the niacin equivalents (NE) (1 mg niacin = 1 mg NE = 60 mg dietary tryptophan). ^f^ DFE: dietary folate equivalents. For the combined intakes of food folate and folic acid, DFEs can be computed as follows: μg DFE = μg food folate + (1.7 × μg folic acid). ^g^ As the dietary folate equivalents (DFE). Furthermore, 1 DFE = 1 μg of folate from food = 0.6 μg of folic acid from fortified foods or from supplements taken with food = 0.5 μg of folic acid from supplements was taken on an empty stomach. ^h^ RE: retinol equivalents, 1 μg RE equals 1 μg of retinol, 6 μg of β-carotene, and 12 μg of other provitamin A carotenoids. ^i^ As retinol activity equivalents (RAEs). 1 RAE = 1 μg of retinol, 12 μg of β-carotene, 24 μg of α-carotene, or 24 μg of β-cryptoxanthin. ^j^ Assuming minimal cutaneous synthesis. In the presence of an endogenous cutaneous synthesis of vitamin D, the dietary vitamin D requirements are lower or even zero. Further, 1 μg of vitamin D ingested = 40 International Units (IU) and 0.025 μg of vitamin D ingested = 1 IU. ^k^ As cholecalciferol. ^l^ Such as ∝-tocopherol, which includes RRR-∝-tocopherol, the only form naturally present in foods, as well as the synthetic 2R-isomeric forms of ∝-tocopherol, which are found in certain fortified foods and supplements. ^m^ Depending on the level of a phytate intake; the higher the phytate intake, the higher the zinc requirement. Abbreviations: EFSA, European Food Safety Authority; PRI, population reference intake; AI, adequate intake; IOM, Institute of Medicine; RDA, recommended dietary allowances; Kcal, kilocalories; ALAP: as low as possible, while consuming a nutritionally adequate diet; PUFAs, polyunsaturated fatty acids; SFA, saturated fatty acids; MUFAs, monounsaturated fatty acids; EPA, eicosapentaenoic acid; DHA, docosahexaenoic acid; MJ: megajoule; and y, years.

**Table 6:** Prevalence of inadequate intakes of specific nutrients in omnivore human milk donors with full-term infants (Donors) and in vegetarian/vegan lactating mothers (Veg) ^1^.

Nutrient	H-AR * [50]	Donors (*n* = 92), *n* (%)	Veg (*n* = 20), *n* (%)	*p* Value
Thiamine (B_1_), mg	1.2	5 (5.4%)	0 (0.0%)	0.286
Riboflavin (B_2_), mg	1.7	13 (14.1%)	9 (45%)	0.002
Niacin (B_3_), mg	13	0 (0.0%)	0 (0.0%)	-
Pantothenic acid (B_5_), mg	5.6	22 (23.9%)	6 (30%)	0.568
Pyridoxine (B_6_), mg	1.4	1 (1.1%)	0 (0.0%)	0.640
Biotin (B_7_), μg	36	34 (37.0%)	7 (35.0%)	0.869
Folate food + folic acid (B_9_), μg	380 (DFE)	36 (39.1%)	0 (0.0%)	<0.001
Cobalamin (B_12_), μg	2.4	0 (0.0%)	5 (25.0%)	<0.001
Vitamin C, mg	145	34 (37.0%)	4 (20.0%)	0.147
Vitamin A, μg RAE	1020	32 (34.8%)	6 (30.0%)	0.682
Vitamin D, μg	10	81 (88.04)	15 (75.00)	0.131
Vitamin E, mg	16	45 (48.9%)	5 (25.0%)	0.051
Iodine, μg	209	40 (43.5%)	8 (40.0%)	0.776
Calcium, mg	860 (19–30 y) 750 (31–50 y)	6 (6.5%)	9 (45.0%)	<0.001
Phosphorous, mg	580	0 (0.0%)	0 (0.0%)	-
Selenium, μg	59	1 (1.1%)	0 (0.0%)	0.640

^1^ The number and percentage of women with inadequate intakes of each nutrient (below harmonized average requirements) are presented in each group. * The H-AR, the harmonized average requirement, was proposed by Allen et al., (2020) [50], after they selected values from the standards set by EFSA (for Europe) and the IOM (for the United States and Canada), giving priority to those published most recently. Abbreviations: DFE, dietary folate equivalents; RAE, retinol activity equivalents; and y, years.

**Table 7:** Diet survey: five-day dietary diaries. Number of food servings per day consumed by the participants, healthy eating index (HEI), and records of supplement and iodized salt intake of the omnivore human milk donors with full-term infants (Donors) and vegetarian/vegan lactating mothers (Veg).

	Donors (*n* = 92)	Veg (*n* = 20)	*p* Value	Recommendations [24] ^a^
Servings per day:				
Dairy	2.58 (0.13)	0.58 (0.28)	<0.001	≥4 ^b^
Grains, legumes, and nuts	6.12 (0.20)	8.11 (0.60)	0.003	≥7
Vegetables and greens	3.40 (0.14)	5.11 (0.33)	<0.001	≥4
Fruits	1.85 (0.13)	1.87 (0.22)	0.654	≥3
Eggs, meat, and fish	2.94 (0.11)	0.28 (0.05)	<0.001	2–3 ^c^
HEI	63.26 (0.92)	61.69 (1.50)	0.478	
Supplement intake: yes, *n* (%)	51 (55.4%)	17 (85%)	0.028	
Iodized salt intake: yes, *n* (%)	62 (67.4%)	14 (70.0%)	0.359	
Salt (g/day)	1.10 (0.06)	1.89 (0.26)	<0.001	

The quantitative variables are presented as means (standard error of the mean). The qualitative variables are expressed as the absolute and relative frequencies (%). ^a^ Number of recommended daily servings of food for lactating women. ^b^ Preferably skimmed or semi-skimmed. ^c^ Preferably fat-free or very low-fat. Abbreviations: HEI, Health Eating Index.

**Table 8:** Diet survey: food consumption frequency questionnaire (FFQ, the number of food servings per day or week that were consumed by the participants) of the omnivore human milk donors with full-term infants (Donors) and vegetarian/vegan mothers (Veg).

	Donors (*n* = 92)	Veg (*n* = 20)	*p* Value	Serving Size [17] ^a^
Milk (servings/day)	1.48 (0.11)	0.26 (0.17)	<0.001	200–250 mL
Other dairy products (servings/day)	1.06 (0.10)	0.21 (0.15)	<0.001	Yogurt 200–250 g Fresh cheese 80–125 g Cured cheese 40–60 g
Meats and derivatives (servings/day)	0.73 (0.06)	0.00 (0.00)	<0.001	100–125 g
Fish (servings/week)	2.19 (0.14)	0.01 (0.01)	<0.001	125–150 g
Eggs (servings/week)	2.92 (0.18)	1.07 (0.40)	<0.001	60 g
Fruits (servings/day)	2.22 (0.16)	2.87 (0.44)	0.146	120–200 g
Raw vegetables (servings/day)	0.64 (0.06)	1.04 (0.15)	0.002	150–200 g
Cooked vegetables (servings/day)	0.80 (0.05)	1.43 (0.15)	<0.001	150–200 g
Legumes (servings/week)	1.39 (0.09)	5.03 (0.55)	<0.001	60–80 g
Bread (servings/day)	1.79 (0.18)	1.73 (0.34)	0.959	40–60 g
Pasta, rice, other grains (servings/week)	3.31 (0.35)	7.28 (1.20)	<0.001	60–80 g
Nuts (servings/week)	4.14 (0.68)	8.32 (1.15)	0.003	25 g
Oils and fats (servings/day)	2.84 (0.30)	2.27 (0.33)	0.765	10 g
Sweets (grams/week)	285.83 (44.06)	124.36 (22.34)	0.053	

^a^ Serving weights for each food group. Data are presented as means (standard error of the mean).

**Table 9:** Erythrocytes and plasma fatty acid composition (g/100 g of total fat) of the omnivore human milk donors with full-term infants (Donors) and vegetarian/vegan lactating mothers (Veg).

Fatty Acid (%)	Common Name	Donors (*n* = 92)	Veg (*n* = 20)	*p* Value
ERYTHROCYTES				
Saturated Fatty Acids (SFAs)				
C14:0	Myristic	0.12 (0.00)	0.10 (0.01)	0.041
DMA C16:0	Dimethylacetal C16:0	2.20 (0.02)	1.64 (0.05)	<0.001
C16:0	Palmitic	21.20 (0.21)	19.97 (0.40)	0.022
DMA C18:0	Dimethylacetal C18:0	3.46 (0.03)	2.92 (0.13)	<0.001
C18:0	Stearic	20.25 (0.15)	19.40 (0.34)	0.066
C24:0	Lignoceric	2.40 (0.09)	3.65 (0.22)	<0.001
Monounsaturated Fatty Acids (MUFAs)				
C17:1	Margaroleic	0.35 (0.01)	0.52 (0.04)	<0.001
C18:1 *cis*-11 (n7)	Cis vaccenic	0.24 (0.01)	0.32 (0.03)	0.001
C18:1 *cis*-9 (n9)	Oleic	12.62 (0.15)	13.40 (0.38)	0.007
*n*-6 Polyunsaturated Fatty Acids (*n*-6 PUFAs)				
C18:2 (n6)	Linoleic	8.12 (0.14)	9.24 (0.31)	0.002
C20:3 (n6)	Dihomo-γ-linolenic	0.99 (0.05)	1.57 (0.14)	<0.001
C20:4 (n6)	Arachidonic	24.30 (0.32)	24.91 (0.75)	0.130
*n*-3 Polyunsaturated Fatty Acids (*n*-3 PUFAs)				
C20:5 (n3)	Eicosapentaenoic	0.12 (0.02)	0.00 (0.00)	0.009
C22:5 (n3)	Docosapentaenoic	0.73 (0.03)	0.66 (0.04)	0.151
C22:6 (n3)	Docosahexaenoic	2.90 (0.12)	1.72 (0.32)	<0.001
Fatty Acid Families				
DMAs		5.66 (0.05)	4.55 (0.16)	<0.001
SFAs		43.98 (0.28)	43.12 (0.49)	0.347
MUFAs		13.20 (0.15)	14.23 (0.44)	0.003
PUFAs		37.09 (0.39)	38.10 (0.65)	0.308
MCFAs (C8-C15)		0.12 (0.00)	0.10 (0.01)	0.041
LCFAs (C16-C18)		62.78 (0.46)	62.85 (0.73)	0.924
VLCFAs (C20-C24)		29.04 (0.39)	28.85 (0.76)	0.992
*n*-6 PUFAs		33.41 (0.30)	35.72 (0.59)	0.007
*n*-3 PUFAs		3.75 (0.16)	2.38 (0.35)	<0.001
*n*-6 PUFAs/*n*-3 PUFAs		10.83 (0.59)	21.09 (2.49)	<0.001
PLASMA				
Saturated Fatty Acids (SFAs)				
C14:0	Myristic	0.28 (0.01)	0.24 (0.03)	0.062
C15:0	Pentadecylic	0.05 (0.00)	0.03 (0.00)	0.003
DMA C16:0	Dimethylacetal C16:0	0.20 (0.01)	0.12 (0.01)	<0.001
C16:0	Palmitic	21.17 (0.17)	19.10 (0.49)	<0.001
DMA C18:0	Dimethylacetal C18:0	0.08 (0.00)	0.05 (0.01)	<0.001
C18:0	Stearic	6.26 (0.07)	5.86 (0.15)	0.033
Monounsaturated Fatty Acids (MUFAs)				
C16:1 *cis*-9 (n7)	Palmitoleic	0.43 (0.02)	0.42 (0.08)	0.010
C18:1 *cis*-11 (n7)	Cis vaccenic	0.44 (0.01)	0.55 (0.04)	0.002
C18:1 *cis*-9 (n9)	Oleic	18.40 (0.26)	19.92 (0.99)	0.411
*n*-6 Polyunsaturated Fatty Acids (*n*-6 PUFAs)				
C18:2 (n6)	Linoleic	39.20 (0.46)	41.28 (1.42)	0.390
C20:3 (n6)	Dihomo-γ-linolenic	1.11 (0.06)	1.43 (0.18)	0.057
C20:4 (n6)	Arachidonic (ARA)	11.30 (0.33)	10.56 (0.71)	0.533
*n*-3 Polyunsaturated Fatty Acids (*n*-3 PUFAs)				
C20:5 (n3)	Eicosapentaenoic (EPA)	0.24 (0.03)	0.08 (0.02)	0.012
C22:6 (n3)	Docosahexaenoic (DHA)	0.83 (0.06)	0.37 (0.07)	<0.001
Fatty Acid Families				
DMAs		0.29 (0.01)	0.17 (0.01)	<0.001
SFAs		27.76 (0.19)	25.23 (0.48)	<0.001
MUFAs		19.27 (0.26)	20.89 (1.06)	0.454
PUFAs		52.68 (0.34)	53.71 (1.11)	0.309
MCFA (C8-C15)		0.33 (0.02)	0.27 (0.03)	0.031
LCFA (C16-C18)		85.91(0.40)	87.12 (0.75)	0.361
VLCFA (C20-C24)		13.48 (0.39)	12.44 (0.74)	0.456
*n*-6 PUFAs		51.61 (0.34)	53.26 (1.10)	0.173
*n*-3 PUFAs		1.07 (0.08)	0.45 (0.09)	<0.001
*n*-6 PUFAs/*n*-3 PUFAs		82.35 (6.70)	173.23 (26.35)	<0.001

Data expressed as means (standard error of the mean). Abbreviations: DMA, dimethylacetal, MCFA, medium-chain fatty acids; LCFA, long-chain fatty acids; and VLCFA: very-long-chain fatty acids.

**Table 10:** Erythrocytes, plasma, and urine concentrations of nutrients and biochemical determinations of the omnivore human milk donors with full-term infants (Donors) and vegetarian/vegan mothers (Veg).

Variable ^1^		Donors (*n* = 92)		Veg (*n* = 20)	*p* Value	Comments about the Reference Values or Studies in which the Corresponding Vitamers are Determined ^3^
	*n*	Value ^2^	*n*	Value ^2^		
ERYTHROCYTES						
Hemoglobin (Drabkin colorimetric method)						
g/dL	92	25.57 (0.38)	20	23.33 (0.84)	0.011	
EGRAC (assay kit)	74		7			Normal: <1.2 (Graham, 2005; Berger, 2022) [51,52]
		1.25 (0.03)		1.53 (0.12)	0.029	
Riboflavin insufficiency/deficiency (EGRAC ≥ 1.4) [26,51,52]		22 (29.7%)		4 (57.1%)	0.224	
Marginal riboflavin status (EGRAC 1.2 to <1.4) [51,52]		20 (27.0%)		2 (28.6%)		
Acceptable riboflavin status (EGRAC < 1.2) [51,52]		32 (43.2%)		1 (14.3%)		
Riboflavin, B2 (UPLC-MS/MS)	92		20			Mean (SE): 141 (4) nM, range: 133–149 nM. A total of 84 Nepali women in their first to seventh month of pregnancy; HPLC (Graham, 2005) [51] Only trace amounts (<1 nM); a total of 124 healthy elderly subjects (mean age of 69 years) recruited, with 38 males and 86 females; Northern Ireland; electrophoresis with fluorescence detection (Hustad, 2002) [53]
ng/L		767.92 (37.71)		932.02 (119.42)	0.277	
nM		2.04 (0.10)		2.48 (0.32)		
ng/g Hb		3.09 (0.16)		4.12 (0.55)	0.115	
Riboflavin deficiency (<170 nM) [51]		92 (100%)		20 (100%)	-	
Nicotinamide, B3 (UPLC-MS/MS)						No data
mcg/L		4718.23 (298.58)		5039.97 (636.03)	0.659	
mcM		38.64 (2.44)		41.27 (5.21)		
mcg/g Hb		18.51 (1.28)		22.41 (3.06)	0.327	
Pantothenic acid, B5 (UPLC-MS/MS)	92		20			No data
mcg/L		58.00 (9.32)		111.85 (35.59)	0.849	
nM		264.56 (42.51)		510.19 (162.34)		
mg/g Hb		233.36 (42.00)		489.65 (154.86)	0.964	
Pyridoxamine, B6 (UPLC-MS/MS)	92		20			No data
mcg/L		519.43 (23.59)		612.83 (33.22)	0.044	
mcM		3.07 (0.14)		3.62 (0.20)		
mcg/g Hb		2.06 (0.11)		2.73 (0.20)	0.006	
PLASMA						
Thiamin, B1 (UPLC-MS/MS)	92		20			Median (25th; 95th percentiles) 2.09 (1.02; 4.06) nM. A total of 1150 Australian adult women aged 43.6 (4.8) years; UHPLC/MS-MS (Andraos, 2021) [54] Reference range: 4–15 nM (Ehsanian, 2020) [55] Mean (SD): 11.4 (3.6) nM. A total of 24 healthy controls (12 males and 12 females), 68.1 (6.8) years old; Sweden; LC-MS/MS (Håglin, 2020) [56] Range: 8–32 nM. A total of 15 adults; USA; LC-MS (Khaksari, 2017) [57] Mean (5th; 95th percentiles): 1.6 (0.3; 14) nM. A total of 196 healthy, non-pregnant, non-lactating Cambodian women, aged 18–45, at baseline time; HPLC-MS/MS (McCann, 2017) [58] Median (25th, 95th percentiles): 6.82 (4.47, 7.02) nM. A total of 21 healthy control children (15 females and 6 males), 8.3 ± 2.1 (range 5–12) years old; Italy; HPLC with fluorimetric detection (Anwar, 2016) [59] Median (ranges): 2.4 (0–4.4) nM pre-intervention and 14.2 (9.2–18) nM post-intervention (100 mg thiamine hydrochloride daily for 4 consecutive days) in 16 not previously supplemented healthy Cambodian nursing mothers, aged 21–35 years with the infants’ ages between 1 and 7 months; 14.3 (7.8–20.7) nM in 16 matched healthy American nursing mothers supplemented with 1.5–3 mg thiamine daily prepartum and postpartum; HPLC (Coats, 2013) [60] Mean (SD): 11.3 (3) nM. A total of 3 healthy volunteers aged 25–49 years; Belgium; HPLC (Gangolf, 2010) [61] Mean (SD): 64.1 (12.0) nM. A total of 20 healthy control volunteers (10 males, 10 females) aged 53 ± 10 years; UK; HPLC with fluorometric detection (Thornalley, 2007) [62]
mcg/L		0.42 (0.03)		0.49 (0.10)		
nM		1.24 (0.09)		1.45 (0.30)		
Riboflavin, B2 (UPLC-MS/MS)	92		20			Median (25th; 95th percentiles): 14.56 (9.22; 25.01) nM. A total of 1150 Australian adult women aged 43.6 (4.8) years; UHPLC/MS-MS (Andraos, 2021) [54] Range: 16–63 nM. A total of 15 adults; USA; LC-MS (Khaksari, 2017) [57] Mean (SD), at baseline (not supplemented) in each group: 12.7 (4.8) nM in group 1 (*n* = 12), 10.0 (4.1) nM in group 2 (*n* = 12), 12.4 (3.6) nM in group 3 (*n* = 12), 11.5 (5.6) nM in group 4 (*n* = 13), 11.3 (4.9) nM in group 5 (*n* = 12), 12.5 (4.2) nM in group 6 (*n* = 12). Chinese male adults aged 18–22 years; HPLC (Guo, 2016) [63] Average (range): 17.8 (5.5–61.6) nM. A total of 120 adults not taking vitamin supplements, aged 19–65 years, in United States; reversed-phase HPLC. Reference interval: 6.7–50.1 nM (Petteys, 2011) [64] Mean (SD), at baseline (not supplemented) in each group: 21.97 (25.9) nM in placebo group (*n* = 40), 21.23 (26.53) nM in 2 mg riboflavin group (*n* = 36), and 17.43 (17.18) nM in 4 mg riboflavin group (*n* = 39). Women aged 19–25 years in United Kingdom, with EGRAC > 1.4; HPLC (Powers, 2011) [65] Mean: 15.3 nM; median (10th; 90th percentiles): 10.5 (5.4, 28.4) nM. A total of 118 elderly individuals in Ireland who did not take vitamin B supplements; laser-induced fluorescence (Hustad, 2002) [53]
mcg/L		23.01 (1.86)		14.79 (1.89)	0.006	
nM		61.14 (4.94)		39.30 (5.02)		
Riboflavin < 6.7 nM [64]		0 (0.0%)		0 (0.0%)		
Nicotinamide, B3 (UPLC-MS/MS)	92		20			Median (25th, 95th percentiles): 396.41 (264.47, 644.74) nM. A total of 1150 Australian adult women aged 43.6 (4.8) years; UHPLC/MS-MS (Andraos, 2021) [54] Average (max, min): 1206.5 (275.2, 6005.1) nM. A total of 57 healthy Emirati volunteers (not detected in 3 samples). LC-MS/MS (Ibrahim, 2020) [66] Median (max, min): 180 (80, 470) nM in the initial cohort (*n* = 30) and 210 (90, 260) nM in the replicative cohort (*n* = 15). Individuals (50% women) undergoing cataract surgery in France, aged 55–83 years, control group of patients with open-angle glaucoma; LC-MS/MS (Kouassi Nzoughet, 2019) [67] Range: 160–1300 nM. A total of 15 adults; USA; LC-MS (Khaksari, 2017) [57] Average (max, min): 274.4 (69.1, 479.6) nM. A total of 20 unknown human plasma samples obtained from Innovative Research (King of Prussia, USA); LC-MS (Meisser Redeuil, 2015) [68]
mcg/L		4.77 (0.36)		3.46 (0.27)	0.052	
nM		39.06 (2.95)		28.33 (2.21)		
Pantothenic acid, B5 (UPLC-MS/MS)	92		20			Median (25th, 95th percentiles): 175.96 (138.79, 233.43) nM. A total of 1150 Australian adult women aged 43.6 (4.8) years; UHPLC/MS-MS (Andraos, 2021) [54] Range: 110–740 nM. A total of 15 adults, but only detected in 8; USA; LC-MS (Khaksari, 2017) [57]
mcg/L		175.24 (52.12)		170.05 (35.48)	0.410	
nM		799.30 (237.90)		775.60 (161.80)		
Pyridoxine, B6 (UPLC-MS/MS)	92		20			Mean (SD): 6.29 (1.57) nM; 7 premenopausal women (21–37 years old) recruited from the Washington State University community, at baseline; HPLC with fluorometric detection (Hansen, 2001) [69] Mean (SD): 0.8 (2.1) nM. Detected in plasma of only 3 of the 21 women studied, aged 22.7 ± 1.8 years; USA; reversed-phase HPLC with fluorometric and ultraviolet detection (Driskell, 1991) [70]
mcg/L		139.29 (3.27)		144.12 (7.21)	0.548	
nM		823.32 (19.3)		851.87 (42.62)		
Pyridoxamine, B6 (UPLC-MS/MS)	92		20			Mean (SD): 5.8 (3.6) nM. Detected in plasma of only 2 of the 21 women studied, aged 22.7 ± 1.8 years; USA; reversed-phase HPLC with fluorometric and ultraviolet detection (Driskell, 1991) [70]
mcg/L		264.26 (4.88)		285.48 (7.30)	0.006	
nM		1571.14 (28.88)		1691.36 (43.40)		
Folate, B9 (UPLC-MS/MS)	91		20			Normal range: >13.4–45.3 nM (Sobczyńska-Malefora, 2018) [71]
mcg/L		3.11 (0.45)		5.20 (2.42)	0.758	
nM		7.05 (1.02)		11.55 (5.48)		
Folate status undetermined (6.8–13.4 nM) [71]		26 (28.6%)		5 (25.0%)	0.739	
Folate deficiency (<6.8 nM) [71]		60 (65.9%)		13 (65.0%)		
Cobalamin, B12 (Competitive immunoassay).	92		20			Normal: >221 pM (Allen, 2018) [72]
pM		557.30 (22.46)		519.80 (61.17)	0.276	
B12 depletion (148–221 pM) [72]		1 (1.1%)		2 (10.0%)	0.025	
B12 deficiency (<148 pM) [72]		0 (0.0%)		0 (0.0%)		
Severe B12 deficiency (<75 pM) [72]		0 (0.0%)		0 (0.0%)		
Holotranscobalamin II (Immunoassay ELISA kit)	92		20			Normal range: 40–150 or 40–200 pM (Allen, 2018) [72]
pM		191.52 (7.61)		185.04 (21.41)	0.384	
B12 depletion (Holo-TC II < 35 pM) [73]		0 (0.0%)		0 (0.0%)	-	
Homocysteine (enzymatic assay)	92		20			
mcM		10.37 (0.37)		8.30 (0.78)	0.006	
Homocysteine elevated (>13 mcM) [71,74]		21 (22.8%)		2 (10.0%)	0.198	
Ascorbic acid (HPLC-DAD)	92		20			Mean plasma concentrations in five studies: 30–84 mcM [75]
mcM		51.88 (3.23)		62.14 (3.80)	0.012	
<11 mcM: scurvy [27]		1 (1.1%)		0 (0.0%)	0.640	
Retinol (HPLC with fluorescence and UV detector)	91		20			
mcg/dL		51.35 (1.61)		44.53 (3.74)	0.056	
mcM		1.79 (0.06)		1.55 (0.13)		
Vit A deficiency (Retinol <20 mcg/dL, <0.7 mcM) [76,77]		0 (0.0%)		0 (0.0%)	-	
25(OH), D (UPLC-electrospray ionization/tandem MS)	92		20			
ng/mL		7.37 (0.42)		9.22 (1.29)	0.264	
nM		18.40 (1.05)		23.01 (3.22)		
Risk for vit D inadequacy (25(OH)D 12- < 20 ng/mL; 30- < 50 nM) [30,78,79]		11 (12.0%)		4 (20.0%)	0.044	
Risk for vitamin deficiency (25(OH)D < 12 ng/mL; <30 nM) [30,78,79]		80 (87.0%)		14 (70%)		
1,25(OH)2D (UPLC-electrospray ionization/tandem MS)	92		20			
pg/mL		146.50 (15.29)		271.41 (35.70)	<0.001	
pM		351.65 (36.70)		651.49 (85.69)		
α-tocopherol (HPLC with fluorescence and UV detector)	90		20			α-tocopherol deficiency (<11.6 mcM) in 70% of lactating South Africa (VIH+ vs. non-VIH) women at 24 weeks postpartum; Mean α-tocopherol concentrations decreased significantly over time after delivery; HPLC (Papathakis, 2007) [80]
mcg/dL		287.63 (24.42)		311.37 (56.64)	0.941	
mcM		6.68 (0.57)		7.23 (1.31)		
Vit E deficiency (<500 mcg/dL; <0.5 mg/dL; <11.6 mcM) [27]		80 (89.9%)		16 (80.0%)	0.218	
Severe vit E deficiency (<5.8 mcM) [81]		56 (62.9%)		9 (45.0%)	0.140	
α-tocopherol:total lipids (cholesterol+triacylglycerols)	89		20			Lower limit of the normal range: 1.6–2.4 mcmol:mmol (Dror, 2011) [81]
(mcmol:mmol)		1.32 (0.12)		1.45 (0.29)	0.196	
Ratio < 1.6 mcmol:mmol [81]		70 (78.7%)		13 (65.0%)		
α-tocopherol:cholesterol (mcmol:mmol)	89	1.47 (0.13)	20	1.63 (0.34)	0.457	Lower limits of the normal range: 2.2–2.5 mcmol:mmol (Dror, 2011) [81]
Ratio < 2.2 mcmol:mmol [81]		77 (86.5%)		16 (80.0%)		
γ-tocopherol (HPLC with fluorescence and UV detector)	88		20			
mcg/dL		37.62 (2.16)		52.66 (4.98)	0.003	
Total cholesterol (enzymatic assay)	92		20			
mg/dL		183.01 (3.51)		182.01 (6.03)	0.840	
mM		4.74 (0.09)		4.71 (0.16)		
Hypercholesterolemia (>240 mg/dL) [79]		6 (6.5%)		1 (5.0%)	0.799	
Triacylglycerols (enzymatic assay)	92		20			
mg/dL		48.93 (1.90)		43.26 (2.43)	0.336	
mM		0.55 (0.02)		0.49 (0.03)		
Hypertriglyceridemia (>200 mg/dL) [79]		0 (0.0%)		0 (0.0%)	-	
HDL (enzymatic assay)	92		20			
mg/dL		62.32 (1.08)		69.49 (2.37)	0.008	
mM		1.61 (0.03)		1.80 (0.06)		
Low HDL levels (<40 mg/dL) [79]		1 (1.1%)		0 (0.0%)	0.640	
LDL (enzymatic assay)	92		20			
mg/dL		103.92 (2.70)		105.26 (6.29)	0.790	
mM		2.69 (0.07)		2.73 (0.16)		
High LDL levels (>160 mg/dL) [79]		3 (3.3%)		0 (0.0%)	0.413	
URINE						
Cr (Jaffé colorimetric kinetic method)	92	121.17 (5.67)	20	121.22 (13.23)	0.776	
mg/dL						
Methylmalonic acid (UPLC-MS/MS)	91		20			Normal ranges are laboratory dependent
mg/L		6.17 (0.32)		5.97 (0.79)	0.618	
mcg/mg Cr		5.28 (0.27)		5.38 (0.81)	0.613	
mcmol/mmol Cr		5.06 (0.26)		5.15 (0.78)	0.613	Normal range: 0.0–3.6 mmol/mol (Henjum, 2020) [82]
B12 deficiency marker (Methylmalonic acid/Cr >4 mcg/mg; >3.8 mmol/mol) [73]		55 (61.1%)		12 (60.0%)	0.927	>2 mcmol/mmol good sensitivity and specificity for serum MMA > 400 nmol/L (Boutin, 2020) [42] 4.8 mcmol/mmol for definite cases of vitamin B12 deficiency (Norman and Morrison, 1993) [83]
Iodine (mcg/L) (ICP-MS)	92		20			
Mean (SE) mcg/L		127.71 (7.57)		112.00 (21.47)	0.061	Adequate: median > 100 mcg/L (WHO, 2013) [84]
Median (p25, p75) mcg/L		109.64 (75.80, 159.54) *		66.28 (50.26, 139.36) *		
mcg/mg Cr		0.11 (0.01)		0.10 (0.02)	0.082	
mcg/g Cr		110 (10)		100 (20)		85–220 mcg/g Cr (Ahn, 2020) [85]
Sodium (ICP-MS)	92		20			
mg/L		3365.51 (139.32)		3693.99 (454.86)	0.704	
M		0.15 (0.01)		0.16 (0.02)		
mg/mg Cr		3.07 (0.15)		3.38 (0.38)	0.528	
mmol/g Cr		133.48 (6.52)		146.93 (16.52)		<47 mmol/g: low; 47–114 mmol/g: intermediate; >114 mmol/L: high (Ahn, 2020) [85]
Calcium (ICP-MS)	92		20			
mg/L		105.00 (9.28)		72.02 (14.68)	0.082	
mg/mg Cr		0.09 (0.01)		0.06 (0.01)	0.024	Normal: <0.14 (Foley, 2010) [86]
Phosphorus (ICP-MS)	92		20			
mg/L		1053.17 (52.08)		840.20 (89.73)		
mg/mg Cr		0.88 (0.03)		0.74 (0.06)		Normal range: 0.22–2.17 (Fernández-Ruiz, 2020) [87]

^1^ The units of our results have been converted to the international system. ^2^ Values are presented as the means (standard error of the mean) for quantitative variables and the number of participants (%) for qualitative variables. ^3^ References have been sorted by year of publication. In the absence of clearly established reference values, the results of studies in which the same vitamers have been determined are presented. For the purpose of comparison, the units of some of the cited manuscripts have also been converted to the international system. * In the case of urinary iodine, the median (25th, 75th percentiles) is also shown, as it is the value used to assess the adequacy of iodine intake in a population. Abbreviations: *n*, number of samples; EGRAC, erythrocyte glutathione reductase activity coefficient: EGRA with an excess of flavine adenine dinucleotide (FAD)/EGRA under baseline conditions; UPLC, ultra-performance liquid chromatography; MS/MS, tandem mass spectrometry; SE, standard error; Hb, hemoglobin; SD, standard deviation; UHPLC, ultra-high-performance liquid chromatography; HPLC, high-performance liquid chromatography; LC, liquid chromatography; Holo-TCII, holotranscobalamin II; DAD, diode array detector; UV, ultraviolet; MS, mass spectrometry; Cr, creatinine; ICP, inductively coupled plasma; and M, molar.

**Table 11:** Macronutrient composition (g/100 mL milk), lipid classes’ profile (g/100 g fat), relative composition of phospholipids (g/100 g polar lipids) and molecular species of triacylglycerols content (g/100 g fat) according to their carbon number (CN) in the human milk of the omnivore human milk donors with full-term infants (Donors) and in the vegetarian/vegan mothers (Veg).

Nutrient	Donors		Veg		*p* Value
	*n*	Mean (SE)	*n*	Mean (SE)	
Macronutrients (g/100 mL milk)					
Lipids	84	3.13 (0.17)	18	3.14 (0.34)	0.958
Carbohydrates		7.73 (0.03)		7.76 (0.06)	0.779
Proteins		1.17 (0.03)		1.16 (0.05)	0.533
Lipid classes (g/100 g fat)					
Triacylglycerols	19	95.56 (0.55)	18	95.33 (0.66)	0.832
Diacylglycerols		3.97 (0.49)		4.18 (0.61)	0.808
Monoacylglycerols		0.04 (0.01)		0.03 (0.01)	0.586
Free fatty acids + cholesterol		0.38 (0.05)		0.41 (0.05)	0.543
Polar lipids		0.05 (0.00)		0.06 (0.00)	0.047
Triacylglycerols (g/100 g fat)					
CN24	19	0.01 (0.00)	18	0.01 (0.00)	0.428
CN26		0.10 (0.01)		0.11 (0.01)	0.713
CN28		0.09 (0.01)		0.06 (0.01)	0.038
CN30		0.21 (0.03)		0.13 (0.02)	0.024
CN32		0.31 (0.05)		0.26 (0.04)	0.543
CN34		0.36 (0.07)		0.22 (0.03)	0.301
CN36		0.40 (0.05)		0.38 (0.05)	0.915
CN38		1.61 (0.16)		1.16 (0.10)	0.022
CN40		1.98 (0.12)		2.09 (0.13)	0.533
CN42		2.65 (0.21)		2.72 (0.26)	0.671
CN44		4.95 (0.32)		4.47 (0.37)	0.627
CN46		7.45 (0.35)		6.60 (0.45)	0.346
CN48		10.68 (0.34)		10.62 (0.62)	0.574
CN50		14.83 (0.49)		12.18 (0.52)	0.001
CN52		37.14 (1.11)		32.37 (1.34)	0.011
CN54		17.23 (1.20)		26.64 (1.47)	<0.001
Phospholipids (g/100 g polar lipids)					
Phosphatidylethanolamine	19	24.62 (1.86)	18	30.72 (1.88)	0.048
Phosphatidylcholine		30.55 (1.10)		26.88 (0.67)	0.018
Sphingomyelin		44.83 (2.58)		42.40 (1.82)	0.429

Variables are presented as means (standard error of the mean).

**Table 12:** Human milk fatty acid methyl esters (FAMEs) composition (g/100 g of total fat) of the omnivore human milk donors with full-term infants (Donors) and vegetarian/vegan mothers (Veg).

Fatty Acid (%)	Common Name	Donors (*n* = 88)	Veg (*n* = 20)	*p* Value	Reference Values	
					European [88] ^1^	World [89] ^2^
Saturated Fatty Acids (SFAs)						
C6:0	Caproic	0.11 (0.00)	0.10 (0.00)	0.631	0.08 ± 0.02	0.13 ± 0.47
C8:0	Caprylic	0.18 (0.00)	0.17 (0.01)	0.095	0.22 ± 0.06	0.21 ± 0.22
C10:0	Capric	1.18 (0.03)	1.05 (0.06)	0.018	1.44 ± 0.34	1.37 ± 0.86
C12:0	Lauric	5.38 (0.17)	5.14 (0.36)	0.719	5.46 ± 1.84	5.7 ± 2.81
C14:0	Myristic	6.50 (0.30)	5.45 (0.44)	0.255	6.19 ± 1.93	6.56 ± 3.05
C15:0		0.19 (0.01)	0.08 (0.01)	<0.001		
C15:0 ai	C15:0 anteiso	0.03 (0.00)	0.02 (0.00)	<0.001		
C15:0 i	C15:0 iso	0.04 (0.00)	0.01 (0.00)	<0.001		
C16:0 i	C16:0 iso	0.02 (0.00)	0.01 (0.01)	<0.001		
C16:0	Palmitic	19.64 (0.27)	15.24 (0.52)	<0.001	21.94 ± 2.92	21.5 ± 4.82
C17:0 ai	C17:0 anteiso	0.05 (0.00)	0.01 (0.00)	<0.001		
C17:0 i	C17:0 iso	0.27 (0.01)	0.30 (0.02)	0.102		
C17:0	Margaric	0.20 (0.01)	0.09 (0.01	<0.001		0.31 ± 0.15
C18:0	Stearic	5.83 (0.14)	4.06 (0.22)	<0.001	6.68 ± 1.59	6.36 ± 2.07
C20:0	Arachidic	0.16 (0.01)	0.17 (0.03)	0.733	0.17 ± 0.04	0.23 ± 0.17
Monounsaturated Fatty Acids (MUFAs)						
C14:1 *cis*-9 (n5)	Myristoleic	0.08 (0.00)	0.03 (0.01)	<0.001		
C16:1 *cis*-9 (n7)	Palmitoleic	1.55 (0.05)	1.32 (0.11)	<0.001	2.21 ± 0.64	2.3 ± 0.92
C17:1	Margaroleic	0.07 (0.00)	0.04 (0.00)	<0.001		
∑ C18:1 trans		0.27 (0.02)	0.07 (0.03)	<0.001	0.66 ± 0.35	
C18:1 *cis*-9 (n9)	Oleic	38.31 (0.53)	41.38 (1.22)	0.025	35.59 ± 4.17	32.6 ± 5.84
C18:1 *cis*-11 (n7)	Cis vaccenic	1.59 (0.03)	1.66 (0.05)	0.282	2.38 ± 0.53	
C20:1 (n9)	Gondoic	0.69 (0.06)	1.42 (0.21)	<0.001	0.38 ± 0.12	0.46 ± 0.28
*n*-6 Polyunsaturated Fatty Acids (*n*-6 PUFAs)						
C18:2 (n6)	Linoleic (LA)	15.29 (0.39)	20.02 (1.15)	<0.001	14.00 ± 4.95	15.7 ± 7.15
C20:2 (n6)	Eicosadienoic	0.27 (0.01)	0.32 (0.02)	0.063	0.26 ± 0.07	0.37 ± 0.19
C20:3 (n6)	Dihomo-γ-linolenic	0.33 (0.01)	0.36 (0.04)	0.652	0.31 ± 0.09	0.37 ± 0.18
C20:4 (n6)	Arachidonic (AA)	0.55 (0.02)	0.46 (0.03)	0.012	0.44 ± 0.12	0.50 ± 0.25
*n*-3 Polyunsaturated Fatty Acids (*n*-3 PUFAs)						
C18:3 (n3)	Linolenic (ALA)	0.52 (0.02)	0.61 (0.05)	0.044	0.94 ± 0.55	1.11 ± 1.05
C22:5 (n3)	Docosapentaenoic (DPA)	0.08 (0.01)	0.04 (0.01)	<0.001		
C22:6 (n3)	Docosahexaenoic (DHA)	0.33 (0.02)	0.15 (0.04)	<0.001	0.34 ± 0.35	0.37 ± 0.31
*n*-7 Polyunsaturated Fatty Acids (*n*-7 PUFAs)						
C18:2 c9, t11 (n7)	Rumenic	0.09 (0.01)	0.04 (0.02)			
Fatty Acid Families						
Not identified		0.21 (0.02)	0.18 (0.02)	0.297		
SFAs		39.78 (0.54)	31.90 (0.91)	<0.001	42.23 ± 5.29	42.2 ± 7.73
MUFAs		42.55 (0.56)	45.91 (1.17)	0.017	41.34 ± 4.48	36.3 ± 6.46
PUFAs		17.46 (0.39)	22.00 (1.15)	<0.001	16.43 ± 5.07	21.2 ± 8.18
SCFAs		0.11 (0.00)	0.10 (0.00)	0.654		
MCFAs (C8-C15)		13.59 (0.47)	11.94 (0.82)	0.317		
LCFAs (C16-C18)		83.60 (0.48)	84.81 (0.93)	0.509		
VLCFAs (C20-C24)		2.50 (0.09)	2.96 (0.24)	0.071		
*n*-6 PUFAs		16.44 (0.39)	21.16 (1.16)	<0.001		17.8 ± 7.51
*n*-3 PUFAs		0.93 (0.04)	0.80 (0.06)	0.104		1.88 ± 2.63
*n*-6 PUFAs/*n*-3 PUFAs		20.90 (1.20)	29.32 (2.49)	0.001		
LA/ALA ratio		33.61 (1.64)	36.46 (3.25)	0.272		
ARA/DHA ratio		2.47 (0.19)	6.68 (1.05)	<0.001	1.68 ± 0.89	

Variables are presented as means (standard error of the mean). ^1^ Data are presented as the mean ± standard deviation, from 223 lactating mothers at a lactation stage of 120 ± 5 days. ^2^ Data of the mature milk are presented as the mean ± standard deviation. Abbreviations: SCFAs, short-chain fatty acids; MCFAs, medium-chain fatty acids; LCFAs, long-chain fatty acids; and VLCFAs, very-long-chain fatty acids.

**Table 13:** Vitamins and minerals in milk of omnivore human milk donors with full-term infants (Donors) and vegetarian/vegan mothers (Veg).

Nutrient ^1^	Mature Milk Nutrient Concentration Reference	Donors	(*n* = 92)	Veg	(*n* = 20)	*p* Value
		*n* (o)	Mean (SE)/Median (RIQ)	*n* (o)	Mean (SE)/Median (RIQ)	
Free thiamin, B1 (UPLC-MS/MS) mcg/L	Free thiamin 18.5 [90] Total thiamin 180 [91]	92 (362)	22.01 (0.92)/17.45 (0.50, 30.40)	20 (80)	24.75 (2.35)/22.50 (7.70, 33.95)	0.560
Free riboflavin, B2 (UPLC-MS/MS) mcg/L	Free riboflavin 11.2 [90] Total riboflavin 364 [92]	92 (362)	78.72 (4.93)/43.05 (22.10, 104.50)	20 (80)	89.49 (22.16)/15.70 (8.80, 60.20)	<0.001
Nicotinamide, B3 (UPLC-MS/MS) mcg/L	Nicotinamide 275 [90] Total niacin 2100 [93]	92 (362)	59.43 (2.62)/44.55 (23.60, 79.30)	20 (80)	33.11 (2.51)/27.10 (13.80, 49.10)	<0.001
Pantothenic acid, B5 (UPLC-MS/MS) mcg/L	2500 [94]	92 (362)	2176.31 (25.85)/2230.70 (1763.80, 2538.30)	20 (80)	2305.05 (62.94)/2370.00 (1929.55, 2718.70)	0.034
Pyridoxal, B6 (UPLC-MS/MS) mcg/L	Pyridoxal 96 [90] B6 130 [95]	92 (362)	40.91 (1.12)/37.05 (26.40, 51.70)	20 (80)	48.46 (3.87)/43.35 (23.70, 59.90)	0.233
Folate, B9 (UPLC-MS/MS) mcg/L	80 [95]	92 (362)	19.82 (0.40)/18.95 (14.60, 25.10)	20 (80)	18.79 (1.57)/15.75 (11.25, 21.30)	0.002
Cobalamin, B12 (Competitive immunoassay)		92 (358)		20 (79)		0.007
pM			482.89 (4.11)/479.70 (435.70–527.80)		545.69 (20.49)/510.90 (440.70–566.80)	
mcg/L	0.5 [96]		0.654 (0.01)/0.650 (0.591, 0.715)		0.740 (0.03)/0.692 (0.597, 0.768)	
Vitamin C * (HPLC-DAD)		91 (358)		20 (80)		0.247
mg/dL			6.33 (0.08)/6.20 (5.45, 7.08)		6.50 (0.16)/6.40 (5.63, 7.77)	
mg/L	35–90 [75]		63.30 (0.80)/62.00 (54.50, 70.80)		65.00 (1.60)/64.00 (56.30, 77.70)	
Retinol (HPLC with fluorescence and UV detector)		91 (357)		19 (76)		<0.001
mcg/dL			1130.67 (243.99)/38.80 (24.10, 68.60)		1123.11 (400.04)/200.60 (40.60, 249.35)	
mcg/L	530 [97]		11306.7 (2439.9)/388.0 (241.0, 686.0)		11231.1 (4000.4)/2000.6 (406.0, 2493.5)	
Vitamin D_3_ (UPLC–electrospray ionization/tandem MS)		92 (362)		20 (80)		<0.001
pg/mL			3433.84 (291.29)/1141.75 (340.15, 4345.90)		1102.01 (224.89)/336.05 (123.55, 1345.55)	
mcg/L	0.25–2 [98]		3.43 (0.29)/1.14 (0.34, 4.35)		1.10 (0.22)/0.34 (0.12, 1.35)	
25(OH)D (UPLC–electrospray ionization/tandem MS)		92 (362)		20 (80)		0.084
pg/mL			82.66 (5.57)/46.50 (23.35, 102.60)		100.36 (11.14)/62.70 (30.70, 131.45)	
mcg/L			0.083 (0.006)/0.047 (0.023, 0.103)		0.104 (0.011)/0.063 (0.031, 0.131)	
α-tocopherol (HPLC with fluorescence and UV detector)		91 (357)		19 (76)		0.206
mcg/dL			465.80 (10.85)/433.00 (309.80, 579.70)		432.34 (23.01)/407.25 (279.35, 558.40)	
mg/L	4.6 [99]		4.65 (0.10)/4.33 (3.10, 5.80)		4.32 (0.23)/4.07 (2.79, 5.58)	
γ-tocopherol (HPLC with fluorescence and UV detector)		91 (357)		19 (76)		<0.001
mcg/dL			53.86 (1.36)/47.20 (35.10, 69.39)		77.48 (4.45)/68.45 (47.10, 104.40)	
mg/L	0.45 [90]		0.539 (0.014)/0.472 (0.351, 0.694)		0.775 (0.045)/0.685 (0.471, 0.104)	
Vitamin E (as TE) ** mg/L	5.2 [90]	91 (357)	4.77 (0.11)/4.46 (3.19, 5.94)	19 (76)	4.52 (0.24)/4.27 (2.99, 5.93)	0.330
Iodine (ICP-MS) ppb (mcg/L)	50–100 [95] 100–200 [100,101]	92 (366)	159.22 (5.13)/135.65 (88.30, 197.50)	20 (80)	126.42 (13.37)/95.65 (50.25, 145.65)	<0.001
Calcium (ICP-MS) ppm (mg/L)	200–300 [102]	92 (366)	98.93 (2.87)/91.00 (54.80, 135.60)	20 (80)	83.54 (3.70)/80.00 (60.40, 102.55)	0.093
Phosphorous (ICP-MS) ppm (mg/L)	120–140 [95,103]	92 (366)	131.22 (1.49)/128.85 (111.30, 145.80)	20 (80)	120.02 (2.25)/120.75 (107.40, 134.20)	0.002
Selenium (ICP-MS) ppb (mcg/L)	18 [104]	92 (366)	11.72 (0.25)/10.80 (9.10, 13.30)	20 (80)	9.90 (0.37)/9.45 (7.75, 11.00)	<0.001

The quantitative variables are presented as the means (standard error of the mean) and as medians (interquartile range). The distribution of all variables was non-normal, except for vitamin C, which had a normal distribution. *n* is the total number of women in each group with data for the studied nutrient, and (o) is the total number of observations for each nutrient. The maximum for each nutrient was 368 in the Donors group and 80 in the Veg group as 4 milk samples were collected for each woman. When these values were not reached, it meant that there were losses in the milk collection or analysis. ^1^ For the purpose of comparison, the units of our results were converted to those used in the references for the nutrients in the human milk. * Vitamin C = ascorbic acid + dehydroascorbic acid. ** Vitamin E is calculated as the sum of each vitamer expressed as tocopherol equivalents (TE) (mg) = α-tocopherol (mg) + 0.25 × γ-tocopherol (mg) [105]. Abbreviations: SE, standard error; IQR, interquartile range; UPLC, ultra-performance liquid chromatography; MS/MS, tandem mass spectrometry; HPLC, high-performance liquid chromatography; DAD, diode array detector; UV, ultraviolet; MS, mass spectrometry; TE, tocopherol equivalents; ICP, inductively coupled plasma; ppb, parts per billion; and ppm, parts per million.

## Data Availability

The data presented in this study are available on request from the corresponding author. The data are not publicly available due to privacy issues.
